# AMPKα2 regulates fasting-induced hyperketonemia by suppressing SCOT ubiquitination and degradation

**DOI:** 10.1038/s41598-023-49991-5

**Published:** 2024-01-19

**Authors:** Lingxue Zhang, Yanqiao Lu, Junqing An, Yin Wu, Zhixue Liu, Ming-Hui Zou

**Affiliations:** Center for Molecular and Translational Medicine, Georgia State University, 157 Decatur Street North East, Atlanta, USA

**Keywords:** Molecular biology, Physiology

## Abstract

Ketone bodies serve as an energy source, especially in the absence of carbohydrates or in the extended exercise. Adenosine monophosphate (AMP)-activated protein kinase (AMPK) is a crucial energy sensor that regulates lipid and glucose metabolism. However, whether AMPK regulates ketone metabolism in whole body is unclear even though AMPK regulates ketogenesis in liver. Prolonged resulted in a significant increase in blood and urine levels of ketone bodies in wild-type (WT) mice. Interestingly, fasting AMPKα2^–/–^ and AMPKα1^–/–^ mice exhibited significantly higher levels of ketone bodies in both blood and urine compared to fasting WT mice. BHB tolerance assays revealed that both AMPKα2^–/–^ and AMPKα1^–/–^ mice exhibited slower ketone consumption compared to WT mice, as indicated by higher blood BHB or urine BHB levels in the AMPKα2^–/–^ and AMPKα1^–/–^ mice even after the peak. Interestingly, fasting AMPKα2^–/–^ and AMPKα1^–/–^ mice exhibited significantly higher levels of ketone bodies in both blood and urine compared to fasting WT mice. . Specifically, AMPKα2^ΔMusc^ mice showed approximately a twofold increase in blood BHB levels, and AMPKα2^ΔMyo^ mice exhibited a 1.5-fold increase compared to their WT littermates after a 48-h fasting. However, blood BHB levels in AMPKα1^ΔMusc^ and AMPKα1^ΔMyo^ mice were as same as in WT mice. Notably, AMPKα2^ΔMusc^ mice demonstrated a slower rate of BHB consumption in the BHB tolerance assay, whereas AMPKα1^ΔMusc^ mice did not show such an effect. Declining rates of body weights and blood glucoses were similar among all the mice. Protein levels of SCOT, the rate-limiting enzyme of ketolysis, decreased in skeletal muscle of AMPKα2^–/–^ mice. Moreover, SCOT protein ubiquitination increased in C2C12 cells either transfected with kinase-dead AMPKα2 or subjected to AMPKα2 inhibition. AMPKα2 physiologically binds and stabilizes SCOT, which is dependent on AMPKα2 activity.

## Introduction

The term “ketone body” is inclusive of three small, lipid-derived molecules: beta-hydroxybutyrate (BHB), acetoacetate, and acetone. In general, the levels of ketone bodies in the body are mainly represented by BHB^1^. During periods of carbohydrate deprivation or prolonged exercise, ketone bodies become an important fuel source for human tissues, including skeletal muscle, brain, and heart^2^. Fasting can lead to elevated levels of ketone bodies in healthy individuals^3^, while prolonged fasting can result in severe hyperketonemia and ketoacidosis^4^. Typically, normal serum levels of ketone bodies are around 0.5 mM, and hyperketonemia is defined as ketone body levels exceeding 1.0 mM, while ketoacidosis is defined as ketone body levels surpassing 3.0 mM^5^. Pathological conditions like diabetic ketoacidosis or situations involving delayed food intake can cause ketone body levels to rise as high as 20 mM. Increasing evidence indicates the ketone bodies elicit broad biological effects. For example, ketone bodies can inhibit cytokine production and inflammasome formation in immunocompetent cells^6^. The use of a ketogenic diet to increase ketone body production serves as an adjunct therapy for epilepsy and shows potential as a treatment for various cancers, including lymphoma, melanoma, neuroblastoma, and kidney cancer^7,8^. Additionally, a clinical study showed that ketone bodies generated via a ketogenic diet improve memory in patients with Alzheimer’s disease^9^.

Ketone body metabolism includes ketogenesis and ketolysis. Ketogenesis primarily occurs in the mitochondria of hepatocytes. Ketogenesis involves the transport of fatty acid into the cell and mitochondria and transformation of fatty acids into ketone bodies when carbohydrates are in short supply. Ketolysis occurs in the mitochondria of many extrahepatic organs and involves the conversion of ketone bodies into either energy or fatty acids and cholesterol. To facilitate the transport of ketone bodies across the cell membrane, a specific transport system exists. Monocarboxylate transporter 1 (MCT1) is the predominant isoform responsible for transporting ketone bodies into the heart and skeletal muscle cells^10^. There are three main enzymes involved in the regulation of ketolysis: succinyl-CoA:3-ketoacid CoA transferase (SCOT), acetoacetyl CoA thiolase-1 (ACAT1), and 3-hydroxybutyrate dehydrogenase-1 (BDH1). BHB is re-oxidized into acetoacetate via BHD1, and then SCOT converts acetoacetate into acetoacetyl CoA. The final step of ketolysis is to cleave the acetoacetyl CoA into two molecules of acetyl CoA by ACAT1^11^. SCOT is considered the rate-limiting enzyme in the process of ketolysis. The supply and metabolism of ketone bodies are crucial for maintaining energy homeostasis in the body. During periods of starvation, ketolysis provides approximately 60–70% of the energy supply to the brain^12^.

AMPK is the main sensor of cellular energy status. It exists as a heterotrimeric complex consisting of one catalytic subunit (α subunit) and two regulatory subunits (β and γ subunits)^13^. AMPK plays important roles in lipid and glucose metabolism. The activation of AMPK leads to a decrease in lipogenesis via phosphorylating its substates: sterol regulatory element-binding protein-1c (SREBP1c) and acetyl CoA carboxylase-1 (ACC1)^14–16^. AMPK also plays a key role in lipolysis^14,15,17^, as it regulates lipolysis by phosphorylating hormone-sensitive lipase^18^ and desnutrin/ATGL^19^. AMPK controlled the capacity of fatty acid oxidation through phosphorylation of ACC2^20,21^. Additionally, AMPK promotes glucose uptake through stimulating glucose transporter-4 (GLUT4) via phosphorylating tre-2/USP6, BUB2, cdc16 domain family member-1, and the Akt substrate of 160 kDa^22–24^. AMPK also affects glucose uptake via insulin^25^ and GLUT1^26^. AMPK plays a role in glycogen synthesis evidenced by chronic activation of AMPK caused an increase in glycogen accumulation and knockout of AMPKα2 blocked the AICAR-induced inactivation of glycogen synthase^26–28^ Furthermore, AMPK inhibits gluconeogenesis by inhibiting hepatocyte nuclear factor-4, CREB-regulated transcription coactivator-2, and class IIa histone deacetylases^28–31^. Despite its active involvement in energy homeostasis, the mechanisms through which AMPK regulates ketone metabolism remain elusive.

In this study, we aim to examine the roles of AMPK in maintaining ketone homeostasis and uncover the underlying mechanisms. Here we report that AMPKα2 regulates ketolysis by binding and stabilizing SCOT.

## Results

### Deletion of AMPKα1 and AMPKα2 enhances fasting-induced hyperketonemia

**Figure 1:**
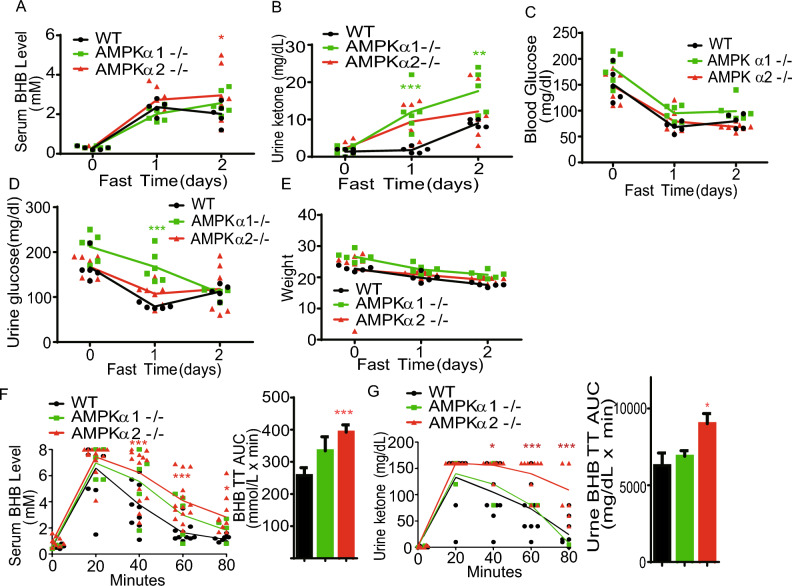
Deletion of AMPKα1 and AMPKα2 enhance fasting-induced hyperketonemia. (**A,B**) Blood BHB (**A**) and urine ketone (**B**) levels in WT, AMPKα1^–/–^, and AMPKα2^–/–^ mice during 2-day fasting (n = 5–7). (**C,D**) Blood (**C**) and urine (**D**) glucose levels in WT, AMPKα1^–/–^, and AMPKα2^–/–^ mice during two-day fasting (n = 5–7). (**E**) Body weights of WT, AMPKα1^–/–^, and AMPKα2^–/–^ mice during 2-day fasting (n = 5–7). (**F,G**) Measurements of blood BHB (**F**) and urine ketone (**G**) levels at different times in WT, AMPKα1^–/–^, and AMPKα2^–/–^ mice after BHB administration. Values represent the mean ± SEM. *P < 0.05; ** P < 0.01; *** P < 0.001 vs. control mice.

To determine the roles of AMPKα1 and AMPKα2 in ketone metabolism, we first measured blood ketone levels in WT, AMPKα1^–/–^, and AMPKα2^–/–^ mice before and during a two-day fasting. As shown in Fig. 1A, AMPKα2^–/–^ mice exhibited a significant elevation in blood BHB levels during fasting compared with WT mice, while AMPKα1^–/–^ mice exhibited no difference. Similarly, AMPKα2^–/–^ mice displayed an approximate 1.5-fold elevation in urine ketone bodies compared to WT mice during fasting (Fig. 1B). However, AMPKα1^–/–^ mice manifested a twofold increase in urine ketone bodies compared to WT mice.

Considering that a decline in blood glucose levels induced by prolonged fasting triggers hepatic ketogenesis, we measured blood glucose and urine glucose levels in fed and fasted WT, AMPKα1^–/–^, and AMPKα2^–/–^ mice. Interestingly, AMPKα1^–/–^ mice exhibited significantly higher concentrations of urine glucose in fasted states (1d) than WT and AMPKα2^–/–^ mice, whereas AMPKα1^–/–^ and AMPKα2^–/–^ mice exhibited the similar blood glucose concentrations as WT mice in both fed and fasted states. However, all three groups—WT, AMPKα1^–/–^, and AMPKα2^–/–^ mice—experienced an equivalent reduction rate in blood glucose levels during fasting, approximately 50% relative to their respective blood glucose concentrations in the fed state at the end of the fasting period (Fig. 1C, D). It is worth noting that the blood glucose levels dropped rapidly during the first day of fasting and remained relatively stable throughout the second day of fasting.

Emerging evidence suggests that insulin exerts a potent inhibitory effect on ketosis by suppressing adipocyte lipolysis and reducing intracellular cyclic adenosine monophosphate (cAMP) levels in hepatocytes^8,32,33^. Then, we measured blood insulin concentrations in fed and fasted WT, AMPKα1^–/–^, and AMPKα2^–/–^ mice and found that blood insulin decreased in both WT and AMPKα2^–/–^ mice but not in AMPKα1^–/–^, mice (data not shown). Furthermore, the fasting insulin levels were higher in AMPKα1^–/–^ mice compared to fasted WT and AMPKα2^–/–^ mice.

Considering that ketone bodies are products of fatty acid oxidation and that lipolysis and intracellular lipid droplet formation are crucial for ketogenesis, we examined blood cholesterol and triglyceride levels during fasting. However, no significant differences in cholesterol and triglyceride levels were observed among WT, AMPKα1^–/–^, and AMPKα2^–/–^ mice during fasting (data not shown). Importantly, the decreases rate of body weights among these mice are the same during the two-day fasting (Fig. 1E). This data indicate that the lipolysis rate should be the similar among these mice.

To explore the roles of AMPKα1 and AMPKα2 in ketolysis, we conducted a BHB tolerance assay in WT, AMPKα1^–/–^, and AMPKα2^–/–^ mice. The results showed that delayed BHB consumption in both AMPKα1^–/–^ and AMPKα2^–/–^ mice compared to WT mice (Fig. 1F). Additionally, the concentration of urine BHB was also higher in AMPKα2^–/–^ mice than in WT and AMPKα1^–/–^ mice (Fig. 1G). Taken together, these data indicate that deletion of AMPKα2 but not AMPKα1 delayed ketone utilization.

### Deletion of skeletal muscle AMPKα2 enhances fasting-induced hyperketonemia

**Figure 2:**
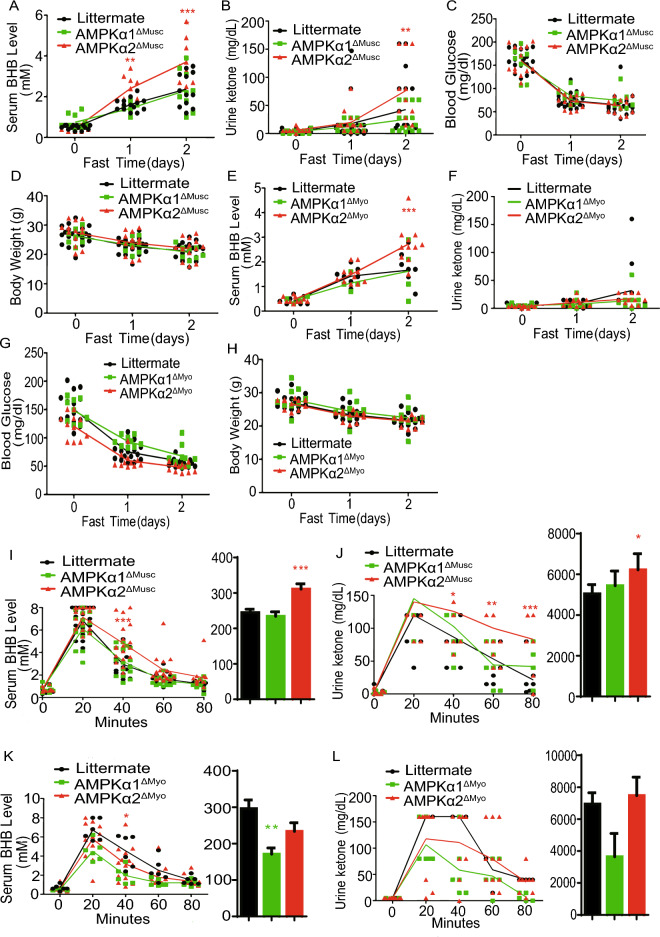
Deletion of skeletal muscle AMPKα2 enhances fasting-induced hyperketonemia. (**A–D**) Blood BHB, urine ketone, blood glucose levels and body weights in littermate, AMPKα1^ΔMusc^, and AMPKα2^ΔMusc^ mice during 2-day fasting. (**E–H**) Blood BHB, urine ketone, blood glucose levels and body weights in littermate, AMPKα1^ΔMyo^, and AMPKα2^ΔMyo^ mice during 2-day fasting (n = 9–17 in each group). (**I–L**) Blood BHB and urine ketone levels in littermate, AMPKα1^ΔMusc^, AMPKα2^ΔMusc^, AMPKα1^ΔMyo^, and AMPKα2^ΔMyo^ mice after BHB administration. Littermate means littermate control mice. Values represent the mean ± SEM. *P < 0.05; **P < 0.01; ***P < 0.001 vs. control mice.

Ketolysis occurs primarily in skeletal muscle, heart, kidney, and brain, but not in liver^34^. Given that skeletal muscle plays a crucial role in maintaining systemic ketone body homeostasis through ketolysis^11^, we further determined the roles of AMPKα1 and AMPKα2 in ketolysis by generating skeletal muscle-specific knockout mice (AMPKα1^ΔMusc^ and AMPKα2^ΔMusc^) and heart-specific knockout mice (AMPKα1^ΔMyo^ and AMPKα2^ΔMyo^).As shown in Fig. 2A, B, both blood and urine ketones increased in all mice during two-day fasting period. However, both blood and urine ketone levels in AMPKα2^ΔMusc^ mice were about twofold higher than in WT and AMPKα1^ΔMusc^ mice after two days fasting. The elevated blood ketones in AMPKα1^ΔMusc^ mice were similar to those in WT mice during fasting, although urine ketone levels in AMPKα1^ΔMusc^ mice were lower than in WT mice after two days fasting (Fig. 2A, B). The decrease rates of blood glucoses and body weights were the same among these mice (Fig. 2C, D). Moreover, compared with WT and AMPKα1^ΔMyo^ mice, AMPKα2^ΔMyo^ mice had a 1.6-fold increase in blood BHB after two days fasting (Fig. 2E). However, AMPKα1^ΔMyo^ and WT mice showed similar increases in blood BHB under fasting conditions. Urine ketone bodies in AMPKα1^ΔMyo^ and AMPKα2^ΔMyo^ mice were lower than their WT littermates after two days fasting (Fig. 2F). In addition, the decreases in blood glucose and body weights levels showed no difference among WT, AMPKα1^ΔMyo^, and AMPKα2^ΔMyo^ mice (Fig. 2G,H).

Similarly, the utilization of ketone bodies in AMPKα2^ΔMusc^ mice was slower than in WT and AMPKα1^ΔMusc^ mice, as evidenced by blood BHB and urine ketone concentration (Fig. 2I,J). Moreover, there were significant difference in the decreasing of blood BHB ketone among WT, AMPKα1^ΔMyo^, and AMPKα2^ΔMyo^ mice in the BHB tolerance assay. However, there were no difference in the decreasing of urine BHB ketone among WT, AMPKα1^ΔMyo^, and AMPKα2^ΔMyo^ mice (Fig. 2K,L).

### AMPKα2 mediates fasting-induced hyperketonemia through SCOT

**Figure 3:**
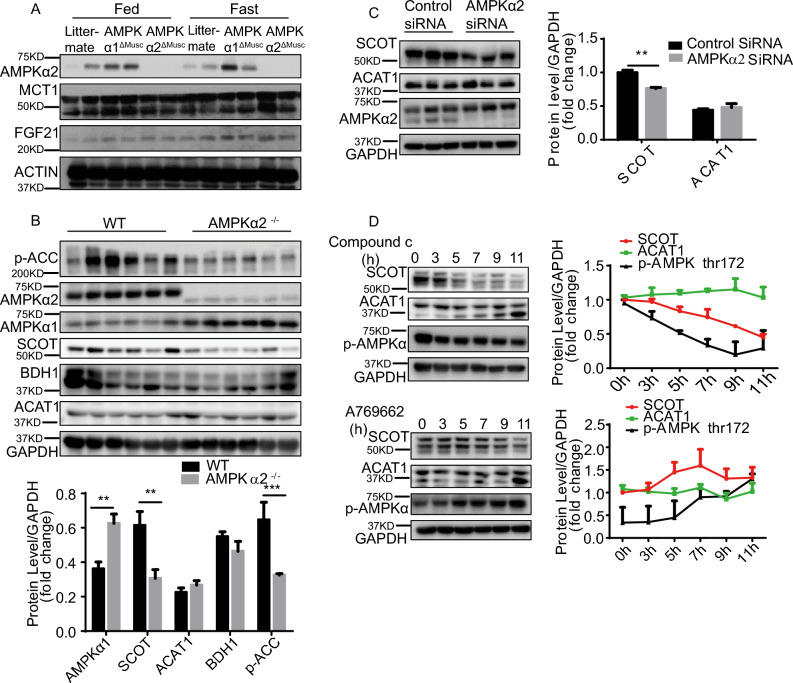
AMPKα2-mediated fasting-induced hyperketonemia is due to the regulation of SCOT expression in skeletal muscle. (**A**) Representative western blot images of MCT1, FGF21, and AMPKα2 expression in skeletal muscle of littermate, AMPKα1^ΔMusc^, and AMPKα2^ΔMusc^ mice after two-day fasting. Beta-actin was used as a loading control. (**B**) Representative western blot images of AMPKα1, AMPKα2, SCOT, BDH1, ACAT1, and p-ACC in skeletal muscle of WT and AMPKα2^–/–^ mice after two-day fasting (n = 6). GAPDH was used as a loading control. (**C**) Determination of SCOT, ACAT1, and AMPKα2 in HEK293T cells transfected with control siRNA and AMPK siRNA and harvested after 48 h (n = 3). GAPDH was used as a loading control. (**D**) Representative western blot images of SCOT, ACAT1, and p-AMPK (T172) in C2C12 cells treated with A769662 (30 µM) and Compound C (10 µM) for 0, 3, 5, 7, 9, and 11 h. GAPDH was used as a loading control. Littermate means littermate control mice. Values represent the mean ± SEM. *P < 0.05; **P < 0.01; ***P < 0.001.

Ketone bodies are absorbed from the blood by peripheral tissues and utilized in extrahepatic mitochondria to regain energy through ketolysis. To determine whether ketone body transport contributes to hyperketonemia in AMPKα2^ΔMusc^ mice, we compared the protein levels of MCT1 among WT, AMPKα1^ΔMusc^, and AMPKα2^ΔMusc^ mice under fed and fasted states (Fig. 3A). The results showed that the deletion of both AMPKα1 and AMPKα2 in skeletal muscle had no effect on the expression of MCT1 under fed and fasted states (Fig. 3A), indicating that ketone transport is not involved in AMPKα2-mediated hyperketonemia. Furthermore, the inhibition of mitochondrial fatty acid oxidation leads to the upregulation of fibroblast growth factor 21 (FGF21) expression in skeletal muscle^35^. To explore the distinct effects of AMPKα1 and AMPKα2 on mitochondrial fatty acid oxidation, we measured FGF21 expression in skeletal muscle of WT, AMPKα1^ΔMusc^, and AMPKα2^ΔMusc^ mice under fed and fasted states. FGF21 expression was increased after fasting, but skeletal muscle AMPKα1 or AMPKα2 did not affect the expression of FGF21 (Fig. 3A).

Excluding the impact of ketone bodies transport on AMPKα2-mediated hyperketonemia, we shifted our focus to ketolysis. We evaluated the effects of SCOT, ACAT1, and BDH1 on AMPKα2-mediated hyperketonemia in skeletal muscle of WT and AMPKα2^–/–^ mice. As shown in Fig. 3B, immunoblot analysis showed that deficient AMPKα2 dramatically reduced SCOT protein expression in skeletal muscle compared to WT mice. However, neither ACAT1 nor BDH1 expression changed in skeletal muscle between WT and AMPKα2^–/–^ mice (Fig. 3B).

To further confirm these findings, we conducted in vitro experiments. The expression of SCOT decreased after AMPKα2 silence in C2C12 cells (Fig. 3C). To determine whether the activity of AMPKα2 influenced the expression of SCOT, we treated C2C12 cells with the A769662 (an AMPK activator) or the Compound C (an AMPK inhibitor). The expression of SCOT decreased after the addition of Compound C (Fig. 3D). But after treatment with A769662, the expression of SCOT shows no significant tendency (Fig. 3D). The expression of ACAT1 did not change upon AMPK inhibition or activation, which is consistent with the in vivo results.

### AMPKα2 stabilizes SCOT by regulating its ubiquitination

**Figure 4:**
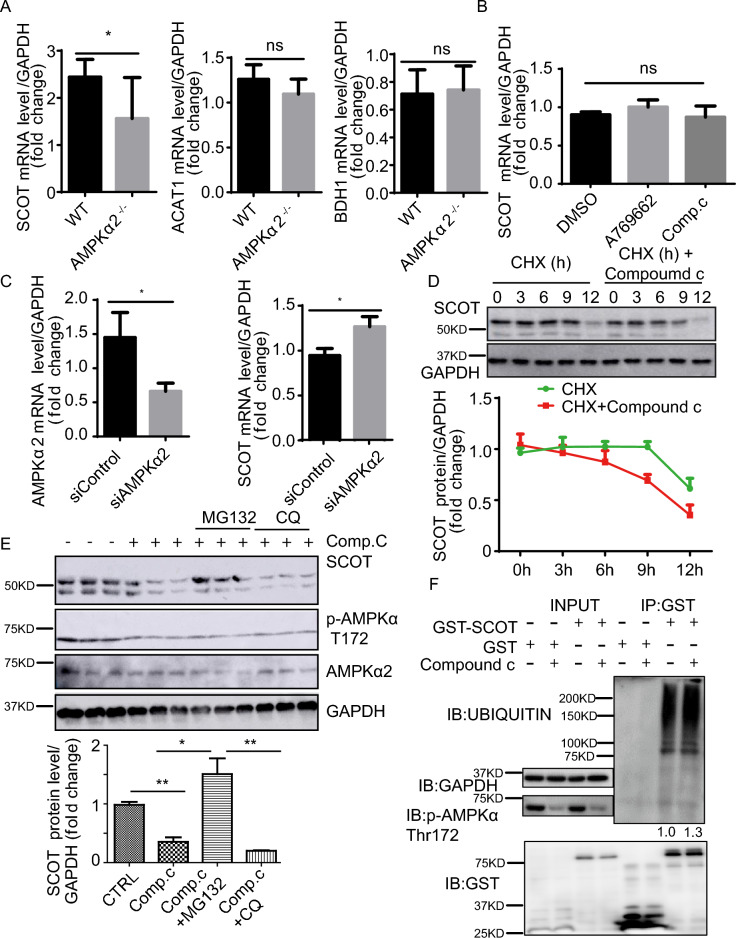
AMPK inhibits SCOT degradation via the proteosome. (**A**) Relative SCOT mRNA levels in skeletal muscle in control and AMPKα2^–/–^ mice after 2-day fasting (n = 8). (**B**) Relative SCOT mRNA levels in C2C12 cells treated with A769662 (30 µM) and Compound C (10 µM) for 6 h (n = 7). (**C**) Relative SCOT mRNA levels in HEK293T cells transfected with control siRNA and AMPKα2 siRNA and harvested after 48 h of transfection (n = 6). (**D**) Representative western blot images of SCOT expression in C2C12 cells treated with CHX (10 µg/mL) and CHX (10 µg/mL) plus Compound C (10 µM) and harvested at 0, 3, 6, 9, and 12 h (n = 2). (**E**) Representative western blot images of SCOT expression in C2C12 cells treated with Comp. C (Compound C,10 µM) or plus MG132 (10 µg/mL) or CQ (10 µg/mL) for 6 h (n = 6). GAPDH was used as a loading control. (**F**) Representative western blot images of ubiquitinated SCOT and p-AMPK (T172) in HEK293T cells transfected with GST-SCOT for 36 h and treated with 10 µM Compound C for 6 h. Glutathione Sepharose beads were used to pull down the GST-SCOT protein (n = 4). Values represent the mean ± SEM. *P < 0.05; ** P < 0.01; *** P < 0.001.

Since AMPKα2 activity regulates SCOT expression, we further explored the mechanism by which this regulation occurs. To elucidate the mechanism by which AMPKα2 regulates SCOT expression, we investigated whether this regulation occurs at the transcriptional level. We measured the mRNA levels of SCOT in the skeletal muscle of WT and AMPKα2^–/–^ mice. As shown in Fig. 4A, SCOT mRNA decreased in vivo followed by the deletion of AMPKα2, while the mRNA levels of ACAT1 and BDH1 remained unchanged. Furthermore, SCOT mRNA levels did not show any significant changes upon AMPK activation or inhibition in C2C12 cells (Fig. 4B). Silencing AMPKα2 in C2C12 cells did not lead to a significant decrease in SCOT mRNA either (Fig. 4C). These findings suggest that the lower SCOT mRNA levels observed in the skeletal muscle of AMPKα2^–/–^ mice may be a consequence of long term disrupted homeostasis resulting from AMPKα2 deletion.

We further investigated whether AMPKα2 regulates the stability of SCOT protein. In C2C12 cells treated with cycloheximide (CHX) to block protein synthesis, we examined the effect of Compound C on SCOT protein levels. We observed that SCOT protein decreased more rapidly in cells treated with both Compound C and CHX compared to cells treated with CHX alone (Fig. 4D). This suggests that AMPKα2 activity regulates the stability of SCOT protein rather than its synthesis. To determine which protein degradation pathway is involved in the regulation of SCOT protein stability by AMPKα2, we treated C2C12 cells with MG132 (a proteasome inhibitor) or chloroquine (CQ, a lysosome inhibitor) in the presence of Compound C. The results showed that MG132 could block Compound C-induced SCOT degradation, but CQ could not (Fig. 4E**)**, suggesting that SCOT is degraded via the proteasome after AMPKα2 inhibition or deletion. Consistent with this result, ubiquitinated SCOT was increased when AMPKα2 was inhibited by Compound C (Fig. 4F). These data demonstrate that AMPKα2 regulates SCOT protein stability via ubiquitination mediated proteasome degradation in skeletal muscle.

### AMPKα2 physiologically binds SCOT and prevents SCOT degradation

**Figure 5:**
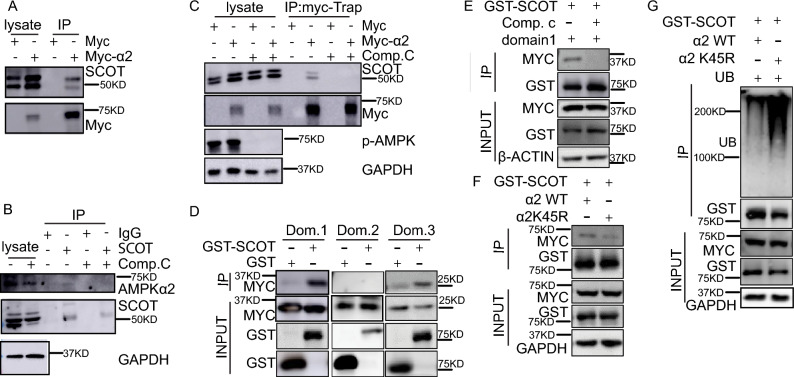
AMPKα2 physiologically binds and stabilizes SCOT. (**A**) Representative western blot images of Myc-AMPKα2 and SCOT in HEK293T cells. HEK293T cells were transfected with Myc or Myc-AMPKα2 for 36 h; Myc-trap agarose was used to immunoprecipate the Myc-tagged protein. (**B**) Representative western blot images of endogenous AMPKα2 and SCOT protein binding of C2C12 cells. C2C12 were cultured to confluent and differentiation for 48 h. Then treated with Compound C (10 µM) for 6 h. The cell lysate in IP buffer and assayed the protein concentration with BCA kit. Total 2 mg proteins for each IP with rabbit IgG or anti-SCOT antibody with Goat anti-rabbit IgG (NEB, S1432) O/N in 4°. The beads were washed and resulted solution for WB with Rabbit true blot anti-rabbit-IgG-HRP (Rockland, 18-8816-31). (**C**) Representative western blot images of Myc-AMPKα2 and SCOT protein. The HEK293 cells were transfected with Myc or Myc-AMPKα2 for 36 h and treated with/out Compound C (10 µM) for 6 h. The cell lysate in IP buffer and assayed the protein concentration with BCA kit. Total 2 mg proteins for each IP with Myc-trap agarose O/N in 4°. The beads were washed and resulted solution for WB with Rabbit true blot anti-rabbit-IgG-HRP (Rockland, 18-8816-31) (**D**) Representative western blot images of AMPKα2 fragments and GST-SCOT in HEK293T cells. HEK293T cells were transfected with MYC-AMPKα2 (domains 1, 2, or 3) and GST or GST-SCOT for 36 h and then glutathione Sepharose beads were used to pull down the GST-SCOT protein. (**E**) Representative western blot images of AMPKα2 domain 1 and GST-SCOT in HEK293T cells. HEK293T cells were transfected with MYC-AMPKα2 domain 1 and GST-SCOT for 36 h and treated with 10 µM Compound C for 6 h. Next, glutathione Sepharose beads were used to pull down the GST-SCOT protein. (**F**) Representative western blot images of AMPKα2 WT, KD AMPKα2, and GST-SCOT in HEK293T cells. HEK293T cells were transfected with MYC-AMPKα2 WT and MYC-KD AMPKα2 for 36 h; glutathione Sepharose beads were then used to pull down the GST-SCOT protein. (**G**) Representative western blot images of ubiquitinated SCOT in HEK293T cells transfected with GST-SCOT, GFP-UB, and MYC-AMPKα2 WT (or KD MYC-AMPKα2) for 36 h; glutathione Sepharose beads were then used to pull down the GST-SCOT protein.

We further explored how AMPKα2 regulates SCOT protein stability. We performed a immunoprecipitation(IP) assay by overexpressing Myc or Myc-AMPKα2 in HEK293T cells. The lysate was IPed with Myc-trap agrose (Proteintech, yta) and the results showed that AMPKα2 binds SCOT (Fig. 5A). Since AMPKα2 is a serine/threonine kinase, we determined whether AMPKα2 could phosphorylate SCOT. However, no detectable phosphorylation of SCOT was observed using a Phos-Tag gel via in vitro kinase assay (data not shown). To test whether AMPKα2 activity affects the interaction between AMPKα2 and SCOT, we performed an immunoprecipitation (IP) assay with endogenous proteins from differentiated C2C12 cells treated with or without Compound C. The results showed that AMPKα2 physically interacted with SCOT and Compound C decreased its interaction (Fig. 5B), suggesting that AMPKα2 activity is crucial for its binding with SCOT. To further confirm Compound C affecting the AMPK and SCOT binding, we perform immunoprecipitation (IP) assay with overexpression of Myc-AMPKα2 in HEK293 cells treated with/out Compound C. The results showed the interaction between SCOT and AMPKα2 was blocked by Compound C (Fig. 5C). To identify the specific binding domains between AMPKα2 and SCOT, we overexpressed three different AMPKα2 truncation mutants : amino acids 13 to 268, the catalytic domain; amino acids 285 to 349, the UBA-like autoinhibitory domain; and amino acids 395 to 550, the regulatory domain. The results showed that only AMPKα2 domains 1 and 3 not domain 2 interacted with SCOT (Fig. 5D). Because the inhibition of AMPKα2 decreased its interaction with SCOT and domain 1 is the catalytic domain, we inhibited domain 1 directly. Figure 5E showed that the inhibition of AMPKα2 domain 1 decreases the binding between AMPKα2 and SCOT, suggesting that the AMPKα2-SCOT interaction depends on the activity of AMPKα2. To further confirm this result, we utilized a kinase-dead (KD) AMPKα2 variant (K45R). The KD AMPKα2 exhibited lower affinity for SCOT compared to the wild-type (WT) AMPKα2 (Fig. 5F). Moreover, overexpression of KD AMPKα2 resulted in a significant increase in the ubiquitination of SCOT, confirming that AMPKα2 regulates the ubiquitination of SCOT (Fig. 5G).

## Discussion

The findings of this study provide novel insights into the role of AMPKα1 and AMPKα2 in regulating fasting-induced hyperketonemia. Our results demonstrate that the deletion of either AMPKα1 or AMPKα2 in mice leads to an increase in fasting-induced hyperketonemia. Both AMPKα1^–/–^ and AMPKα2^–/–^ mice exhibit impaired ketone utilization, as indicated by the BHB tolerance assay. Specifically, we identify that AMPKα2 in skeletal muscle plays a critical role in ketone utilization, as AMPKα2^ΔMusc^ mice exhibit reduced ketone body utilization compared to AMPKα1^ΔMusc^ and WT mice. Notably, the expression levels of MCT1, a ketone body transporter, remain unchanged in the skeletal muscle of littermate control mice, AMPKα2^ΔMusc^ mice and AMPKα1^ΔMusc^ mice. Furthermore, we demonstrate that SCOT levels are reduced in skeletal muscle of AMPKα2^–/–^ mice, whereas the expression levels of ACAT1, and BDH1 are similar both in WT and AMPKα2^–/–^ mice. Additionally, we demonstrate that SCOT protein undergoes degradation in response to AMPKα2 deletion or inhibition in vivo and in vitro*.* Moreover, AMPKα2 physiologically interacts and stabilizes SCOT. Notably, the interaction between AMPKα2 and SCOT relies on the activity of AMPKα2. In summary, our findings highlight the role of AMPKα2 in regulating systemic ketone homeostasis by interacting with and stabilizing SCOT to modulate ketolysis in skeletal muscle.

Our study revealed a significant finding regarding the impact of AMPKα2 and AMPKα1 deletion on hyperketonemia, potentially reaching ketoacidosis levels. Notably, the effects of AMPKα2 deletion were more pronounced than those of AMPKα1 deletion. AMPKα1^–/–^ mice exhibited a slight elevation in blood β-hydroxybutyrate (BHB) levels after fasting, whereas AMPKα2^–/–^ mice showed a much higher elevation compared to WT mice. Interestingly, urine BHB levels in AMPKα1^–/–^ mice were significantly higher than in AMPKα2^–/–^ mice and even exceeded those in WT mice. This observation suggests that AMPKα1^–/–^ mice may experience mild antidiuresis, as evidenced by their lower urinary output compared to WT mice, which could lead to increased water reabsorption in the renal tubules^36^. Antidiuresis leads to increased water reabsorption in the renal tubules. Under conditions that the filtration of renal ketone bodies remains unchanged or increases in AMPKα1^–/–^ mice, the increased reabsorption of water will lead to an increase in urine osmotic pressure and urine ketone levels.

Previous studies have explored the roles of AMPK in ketogenesis and lipolysis. For instance, Marc et al*.*^37^ reported that overexpression of constitutively active AMPKα2 promotes ketogenesis. They demonstrated that AMPKα2 phosphorylates and inactivates ACC, which leads to reduced levels of malonyl CoA, relieving the inhibition of carnitine palmitoyltransferase 1 and allowing mitochondrial fatty acid transport and oxidation in various tissues. Additionally, AMPK can regulate peroxisome proliferator-activated receptor-alpha (PPARα) activity, which is one of the key transcription factors involved in the regulation of ketogenesis. The evidence showed that hepatic fatty acid oxidation and ketogenesis decreased in PPARα-knockout mice during fasting, followed by rapid onset of hypoglycemia^8^. AMPK also functions as a negative regulator of mechanistic target of rapamycin complex 1 (mTORC1). Inadequate levels of liver-specific tuberous sclerosis 1, an mTORC1 inhibitor, lead to a pronounced defect in ketogenesis^38^. However, the precise role and mechanism of AMPK in ketogenesis require further investigation in future studies. AMPK is also involved in the regulation of lipolysis, which provides substrates for ketogenesis. The previous reports showed that AMPK regulates ATGL and HSL activities by direct phosphorylation^18,39^. In cultured adipocytes, AMPK promotes ATGL activity^15^ and blocks HSL activity. In an adipose tissue specific AMPKα1/2 double knockout mouse model^19^, adipose lipolysis was overall enhanced. We did not see differences in lipolysis in our individual AMPKα1^–/–^ or AMPKα2^–/–^ mice because the observed increases in triglyceride and cholesterol levels were similar among WT, AMPKα1^–/–^, and AMPKα2^–/–^ mice. It is possible that AMPKα1 and AMPKα2 may possess complementary functions during adipose lipolysis.

Mice with globally inadequate levels of AMPKα1 and AMPKα2 showed delayed ketone consumption. However, among skeletal muscle specific or cardiomyocyte specific AMPKα1 and AMPKα2 knockout mice, only AMPKα2^ΔMusc^ mice exhibited a slowdown in ketolysis. It may be due to AMPKα1 and AMPKα2 have different tissue distributions and abundances. Stapleton et al.^40^ reported that AMPKα2 is less broadly distributed and most abundant in skeletal muscle with lower levels in heart, liver, and kidney. In contrast, AMPKα1 is widely distributed and much less abundant. Since ketolysis occurs in various tissues except the liver. AMPKα1 may regulate ketolysis in many tissues because it is widely distributed. This can explain our observations that ketone utilization delayed in AMPKα1^–/–^ mice not in AMPKα1^ΔMusc^ and AMPKα1^Δmyo^ mice. On the other hand, AMPKα2 predominantly regulates ketolysis in skeletal muscle, which is the major site of ketone utilization.

In addition, we found that SCOT levels decreased following AMPKα2 deletion. SCOT is the main rate-limiting enzyme in mitochondrial ketone utilization and can mediate the production of acetoacetyl CoA. SCOT deficiency is a major defect observed in ketolysis^41^. SCOT deficiency is associated with a persistent state of ketosis, which is a characteristic feature of the condition. Interestingly, we also observed an increase in SCOT protein ubiquitination and subsequent degradation without any significant change in mRNA levels following AMPKα2 deletion in our in vitro experiments. The exact mechanism by which AMPKα2 regulates SCOT protein stability remains unclear. We speculated that AMPKα2 may phosphorylate SCOT because AMPKα2 is a serine/threonine kinase. Unfortunately, we did not demonstrate phosphorylation of SCOT in an in vitro kinase assay using a Phos-Tag electrophoresis. We also explored the possibility of SCOT tyrosine nitration, as suggested by Marcondes et al.^42^, which could potentially inhibit SCOT activity during inflammatory conditions. However, we did not detect any differences in SCOT tyrosine nitration between AMPKα2 activation and inhibition. Finally, we found that AMPKα2 binds SCOT, preventing its degradation by regulating its ubiquitination. This interaction depends on AMPKα2 activity. Notably, both a kinase-dead (KD) form of AMPKα2 and the inhibition of AMPKα2 were found to promote SCOT degradation, further confirming the importance of AMPKα2 in stabilizing SCOT protein.

AMPK plays a crucial role in various physiological processes, and its dysregulation has been implicated in several diseases, including diabetes, cardiovascular diseases, and cancers. The disruption of AMPK function can have implications for ketone metabolism and may contribute to physiological and pathological effects in human diseases. Ketone bodies are not only an energy resource but also signal molecules that may participate in many metabolic diseases. BHB is an endogenous ligand for the niacin receptor GPCR 109A^43^ to inhibit adipose tissue lipolysis^43,44^. BHB has also been found to modulate sympathetic outflow, reduce heart rate, and decrease total energy expenditure by interacting with GPR41^45^. Additionally, BHB inhibits class I histone deacetylases, leading to increased histone acetylation and upregulation of genes involved in resistance to oxidative stress^46^, supporting the neuroprotective function of ketone bodies^47^. BHB can also reduce the acetylation of p53, an essential tumor suppressor, and the expression of genes for p21 and PUMA, downstream components of p53. These effects result in reduced cell growth arrest and apoptosis in cultured cells under p53-activating conditions^48^. Studies have demonstrated the inhibitory effects of ketone bodies on the growth of pancreatic cancer in mouse models^49^. Moreover, in states of sustained ketosis like diabetic ketoacidosis, the expression and activity of SCOT (encoded by the Oxct1 gene), as well as SCOT protein abundance, are diminished in the heart and skeletal muscle of rodents^50–52^. In diabetic patients or in animal models of diabetes, impaired AMPK activity and reduced SCOT expression specifically in skeletal muscle, but not in the heart or kidney, have been observed compared to controls. This decreased SCOT expression exacerbates the severity of diabetic ketoacidosis. Therefore, targeting AMPK activity holds promise as a therapeutic approach for diabetic ketoacidosis and other disorders associated with ketone metabolism.

## Methods

The data, analytical methods, and study materials used in this research will be made accessible to other researchers upon request. Interested individuals can obtain these resources by contacting the corresponding authors, who will facilitate the process of reproducing the results and replicating the experimental procedures.

### Animal study approval

The animal protocol utilized in this study underwent thorough review and received approval from the Institutional Animal Care and Use Committee at Georgia State University. The committee ensures that all ethical considerations and guidelines for animal welfare are strictly adhered to throughout the research process. The study is reported in accordance with ARRIVE guidelines.

### Animals

WT mice (C57BL6), AMPKα1^–/–^ mice, AMPKα2^–/–^ mice, AMPKα1^fl/fl^ mice, AMPKα2^fl/fl^ mice, AMPKα1^ΔMusc^ mice, AMPKα2^ΔMusc^ mice, AMPKα1^ΔMyo^ mice, and AMPKα2^ΔMyo^ mice were used in this study. WT, muscle creatine kinase (CKMM) cre mice, and alpha myosin heavy chain (Myh6) cre mice were obtained from Jackson Laboratories (Bar Harbor, ME). AMPKα1^–/–^ and AMPKα2^–/–^ mice were generated as previously described^53,54^. AMPKα1^fl/fl^ mice and AMPKα2^fl/fl^ mice were kindly provided by Dr. Benoit Viollet. AMPKα1^ΔMusc^ mice and AMPKα2^ΔMusc^ mice were generated by crossing AMPKα1^fl/fl^ mice and AMPKα2^fl/fl^ mice with muscle creatine kinase (CKMM) cre transgenic mice, resulting in skeletal and cardiac muscle deletions of the respective flanked genomes. AMPKα1^ΔMyo^ mice and AMPKα2^ΔMyo^ mice were generated by crossing AMPKα1^fl/fl^ mice and AMPKα2^fl/fl^ mice with alpha myosin heavy chain (Myh6) cre transgenic mice, resulting in a cardiac muscle deletion of the respective flanked genome. If not mentioned, all mice used in this study were 8–12 weeks old male mice.

All mice were housed under controlled environmental conditions, with a temperature of 20 ± 2 °C and a 12-h light/dark cycle. They were provided with standard chow diet and had unrestricted access to water throughout the study. During the 48-h fasting period, the mice were allowed free access to water and were closely monitored to ensure their well-being.

### Antibodies and reagents

Compound C (#866495-64-3), A769662 (SML2578-5MG), cycloheximide (#66-81-9), chloroquine diphosphate salt (#50-63-5), MG132 (#133407-82-6) were from Millipore Sigma (Burlington, MA). Lipofectamine 2000 transfection reagent (#11668-019), Lipofectamine RNAiMAX (#13778-150), Opti-MEM I reduced serum (#31985-088) were from Fisher Scientific (Waltham, MA). AMPKα2 siRNA (h) (sc-38923), anti- AMPKα2 (sc-19129), anti-MCT1 (sc-50325), anti-actin (sc-47778), anti-BDH1 (sc-514413), anti-GAPDH (sc-137179) were from Santa Cruz Biotechnology (Dallas, Texas). Anti-phospho-ACC (Ser79) (#3661), anti-ACAT1 (#44276), anti-phospho-AMPKα (Thr172) (#2535), anti-GST (#2624s), and anti-MYC-tag (#2276s) were from Cell Signaling Technology (Danvers, MA). Anti-SCOT (ab2411125), anti-FGF21 (ab171941) antibody was from Abcam (Cambridge, UK). MYC-AMPKα2 K45R (#15992), GFP-UB (#11939), AMPKα2 (#74447), and MYC-AMPKα2 WT (#15991) were from Addgene (Watertown, WA).

### Cell culture

293 T (ATCC CRL-3216) and C2C12 (ATCC CRL-1772) cell lines were purchased from the American Type Culture Collection (Rockville, MD), maintained in DMEM (MT10013CV, Thermo Fisher Scientific), and supplemented with 10% FBS and 1% PS (100 U/mL penicillin and 100 µg/mL streptomycin). The differentiation of C2C12 cells was achieved by changing normal media to differentiation media (DMEM supplemented with 5% FBS and 1% PS). After 24 h in differentiation media, fused cells are visible. Differentiation media should be changed every 48 h. The GST-SCOT plasmid was cloned into the pRK5-GST vector using *Sal*I and *Not*I restriction enzymes and the primers in Table 1. Plasmids for MYC-AMPKα2 containing domains 1, 2, and 3 were made from the AMPKα2 plasmid using the primers in Table 1. The cells were transfected with plasmids using Lipofectamine 2000 Reagent or siRNA with Lipofectamine RNAiMAX Transfection Reagent from Invitrogen in Opti-MEMReduced Serum Medium following manufacture’s protocols.

**Table 1:** Primers for constructs.

Primer name	NDA sequence 5′–3′
mSCOT-sal-F	TCGTCGACCATGGCGGCTCTCAAACTCCTG
mSCOT-Not-R	CTGCGGCCGCAGTTGAAATCTGCTGCATTGGCATG
hAMPKα2 domain 1-F	TTCGTCGACCATGGCTGAGAAGCAGAAGCAC
hAMPKα2 domain 1-R	AGTGCGGCCGCAATGACGTTAGCATCATAGGAAGG
hAMPKα2 domain 2-F	TTCGTCGACCATGTTTCCTGAAGACCCTTCCT
hAMPKα2 domain 2-R	AGTGCGGCCGCCAGGCCTGGGGGAATATGC
hAMPKα2 domain 3-F	TTCGTCGACCATGAAACCTCATCCAGAAAGGATG
hAMPKα2 domain 3-R	AGTGCGGCCGCGGCTAAAGTAGTAATCAGACTGGCAC

### Western blot and immunoprecipitation

Tissues and cultured cells were homogenized in radioimmunoprecipitation assay lysis buffer (# 24948, Santa Cruz Biotechnology), which contained protease inhibitor cocktail, detergents, and phenylmethylsulfonylfluoride. The cultured cells were homogenized using RIPA buffer with protease inhibitor cocktails and PMSF. The Tissues were homogenized using RIPA buffer with protease inhibitor cocktails and PMSF with Homogenizer (Qiagen tissuelyser II). If the lysate for immunoprecipitation, the cells and tissues were lyzed in cell lysis buffer (50 mM Tris–HCl pH 7.2, 150 mM NaCl, 0.5% NP-40) plus protease inhibitor cocktails and PMSF. The protein concentrations were measured using a bicinchoninic acid assay (BCA, #23225, Pierce Biotechnology, Rockford, IL). Total cell lysates were immunoprecipitated with glutathione Sepharose beads (GE17-0756-01) (overnight, 4 °C), and the immunoprecipitates were washed for four times with 1 ml cell lysis buffer plus protease inhibitor cocktails and PMSF and then subjected to western blot analysis. For western blot analysis, 20–40 µg protein was resolved by sodium dodecyl sulfate polyacrylamide gel electrophoresis, transferred to PVDF membranes (IPVH00010, Millipore Sigma), and incubated with indicated antibodies. The PVDF membranes were developed with GE AI600 RGB GEL Imaging System. The ubiquitinated proteins were analyzed by the ration of total ubiquitin signal to total proteins or to the total GST-fusion protein.

### Reverse transcription-polymerase chain reaction (RT-PCR)

Total RNA was isolated from 293 T cells or skeletal muscle using TRIzol (#15596018, Thermo Fisher Scientific) and reverse-transcribed using the iScript cDNA synthesis kit (#170-8891, Bio-Rad Laboratories, Inc. Hercules, CA). RT-PCR was performed using the iQ SYBR Green Supermix (#720001059, Bio-Rad Laboratories) in the CFX96 real-time system (Bio-Rad Laboratories). Primer sequences are as in Table 2. The RT-PCR results were normalized with Prism 6 software (GraphPad) with methods described in “Statistical analysis” section.

**Table 2:** Primers for RT-PCR.

Primer name	DNA sequence 5′–3′
Mouse GAPDH-F	ATTGTCAGCAATGCATCCTG
Mouse GAPDH-R	ATGGACTGTGGTCATGAGCC
Human GAPDH-F	GGAGCGAGATCCCTCCAAAAT
Human GAPDH-R	GGCTGTTGTCATACTTCTCATGG
Human AMPKα2-F	GACTTCCTTCACAGCCTCATC
Human AMPKα2-R	CGAGCGACTATCAAAGACATACG
Mouse SCOT-F	CATAAGGGGTGTGTCTGCTACT
Mouse SCOT-R	GCAAGGTTGCACCATTAGGAAT
Human SCOT-F	GGGTCCATATCCACGACAACA
Human SCOT-R	GACGTGTCCACCTCTAATCATTG
Mouse ACAT1-F	CAGG-AAGTAAGATGCCTGGAAC
Mouse ACAT1-R	TTCACCCCCTTGGATGACATT
Human ACAT1-F	ATGCCAGTACACTGAATGATGG
human ACAT1-R	GATGC-AGCATATACAGGAGCAA
Mouse BDH1-F	TTCCCCTTCTCCGAAGAGC
Mouse BDH1-R	CCCAGAGGGTGCATCTCATAG
Human BDH1-F	GACAGC-CTAAACAGTGACCGA
Human BDH1-R	GAGCGGACAATCTCCACCA

### Analytical procedures

BHB and blood glucose concentrations were measured using the Precision Xtra and ReliOn Confirm systems, respectively. Urine ketone levels were qualitatively evaluated using Ketostix reagent strips for urinalysis (Bayer, Leverkusen, Germany). Skeletal muscle tissue BHB levels were measured using the BHB colorimetric assay kit (k632-100, Bio Vision, Milpitas, CA). The serum were obtained from blood in blood collecting tubes (BD bioscience) following centrifuge as manufacture’s protocol when the mice were euthanized. The serum insulin levels were measured using an ELISA (ALPCO, Windham, NH). Serum cholesterol levels were measured using a cholesterol assay kit (ab65390, Abcam), and serum triglyceride levels were measured using a triglyceride colorimetric assay kit (#10010303, Cayman Chemical, Ann Arbor, MI).

### BHB tolerance test

BHB tolerance tests were performed as previously described^55^. Briefly, mice were fasted for 5 h in the morning, and blood BHB concentrations were measured. Each animal then received an intraperitoneal injection of 1.5 g BHB/kg body weight (Sigma-Aldrich). Blood BHB levels were determined 20, 40, 60, and 80 min after the injection.

### Statistical analysis

Statistical analysis was performed using Prism 6 software (GraphPad). The data are presented as means ± SD. Differences between more than two groups were analyzed using one-way *ANOVA*, followed by the Newman-Keuls multiple comparison test. Comparisons of different parameters within each group were made using two-way *ANOVA*, followed by the *Bonferroni* posttest. Statistical significance was considered for P values less than 0.05 (Supplementary Information).

### Supplementary Information

Supplementary Information 1.

Supplementary Information 2.

## Data Availability

The datasets used and/or analysed during the current study available from the corresponding author on reasonable request.
