# Supplementary material for: AMPKα2 regulates fasting-induced hyperketonemia by suppressing SCOT ubiquitination and degradation
Source: Sci Rep. 2024 Jan 19;14:1713. doi: 10.1038/s41598-023-49991-5 (PMC10798978; doi:10.1038/s41598-023-49991-5)
Supplement: Supplementary file 1 — Supplementary Information 1. [file 41598_2023_49991_MOESM1_ESM.pdf]

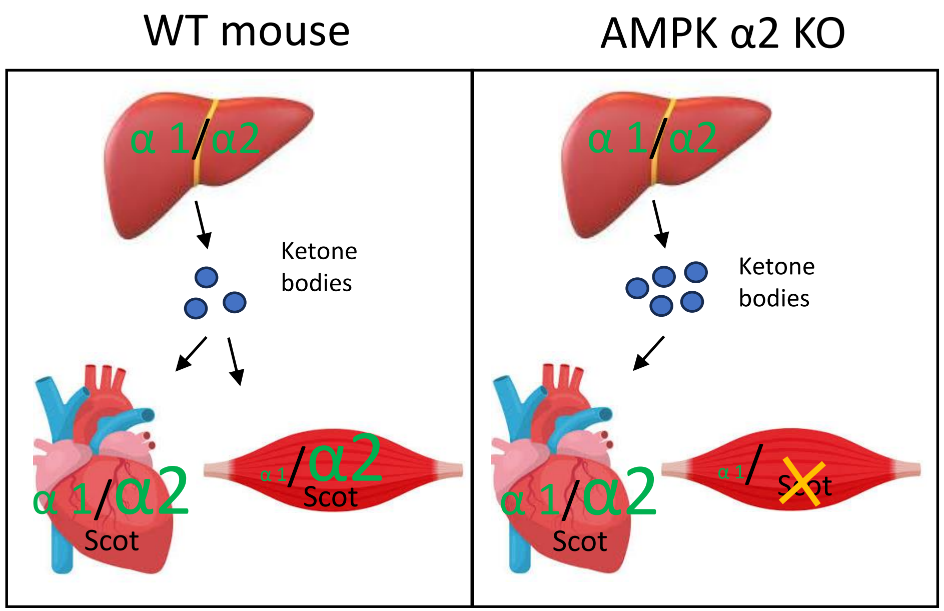

AMPKα2 binds and regulates SCOT activity in mouse tissue.
Ketone accumulated in skeletal muscle specific AMPKα2 KO mice
because AMPKα2 KO decreases Scot activity
