# Supplementary material for: AMPKα2 regulates fasting-induced hyperketonemia by suppressing SCOT ubiquitination and degradation
Source: Sci Rep. 2024 Jan 19;14:1713. doi: 10.1038/s41598-023-49991-5 (PMC10798978; doi:10.1038/s41598-023-49991-5)
Supplement: Supplementary file 2 — Supplementary Information 2. [file 41598_2023_49991_MOESM2_ESM.pdf]

Figure 3A

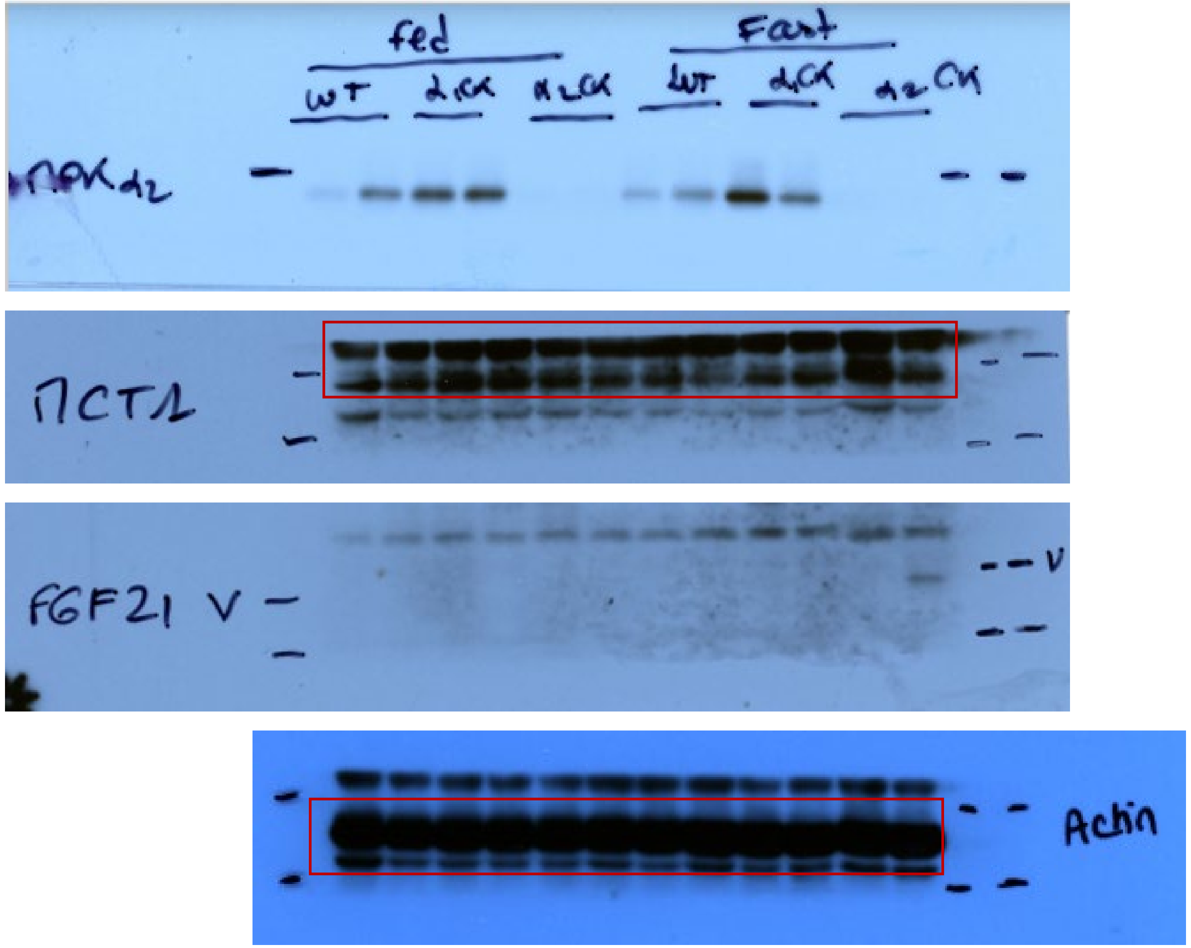

Figure 3B

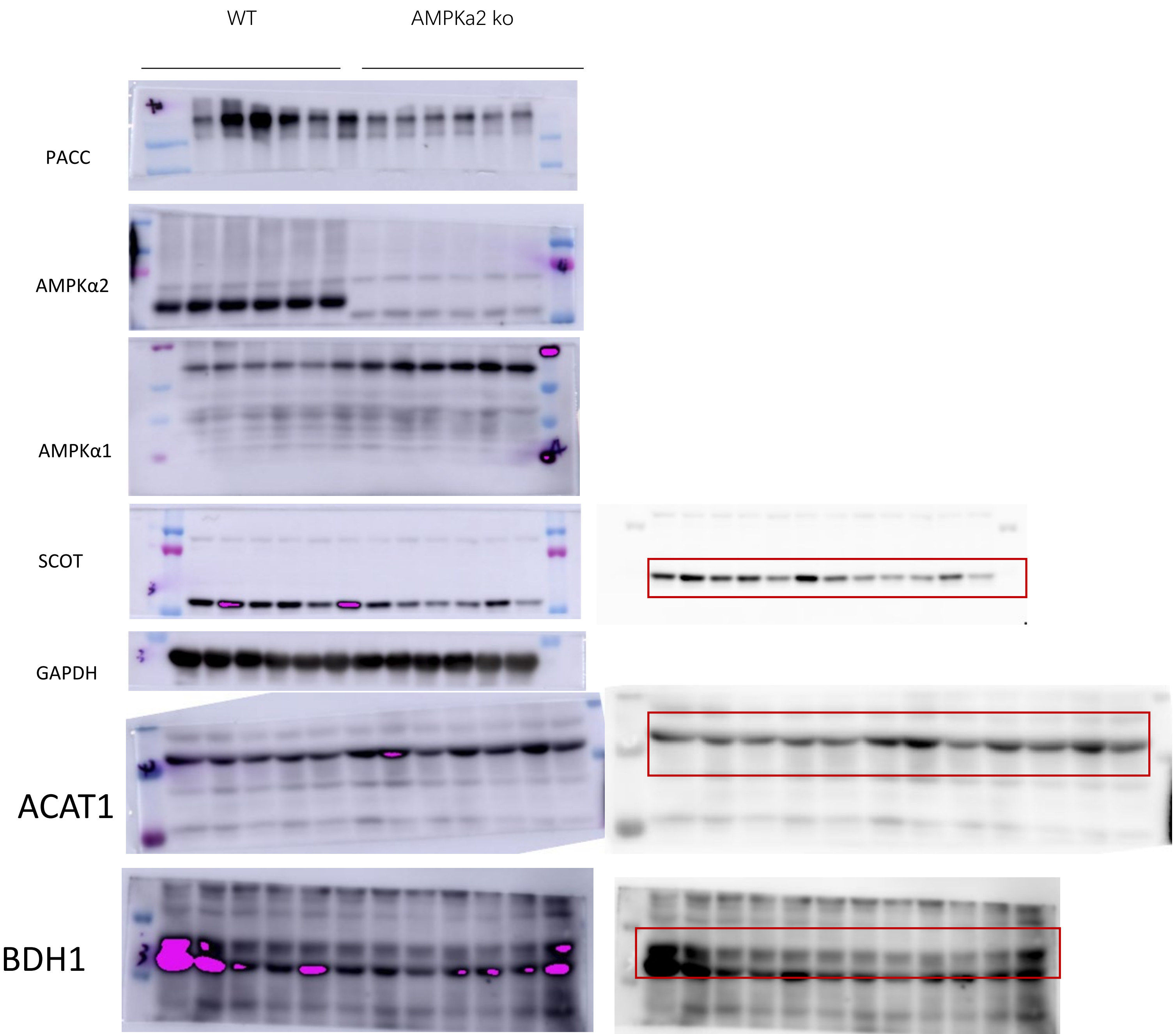

Figure 3C

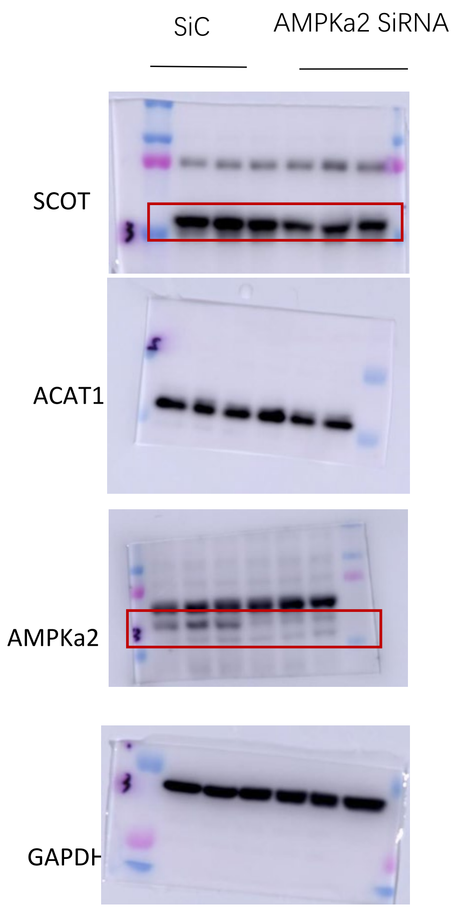

Figure 3D compound c

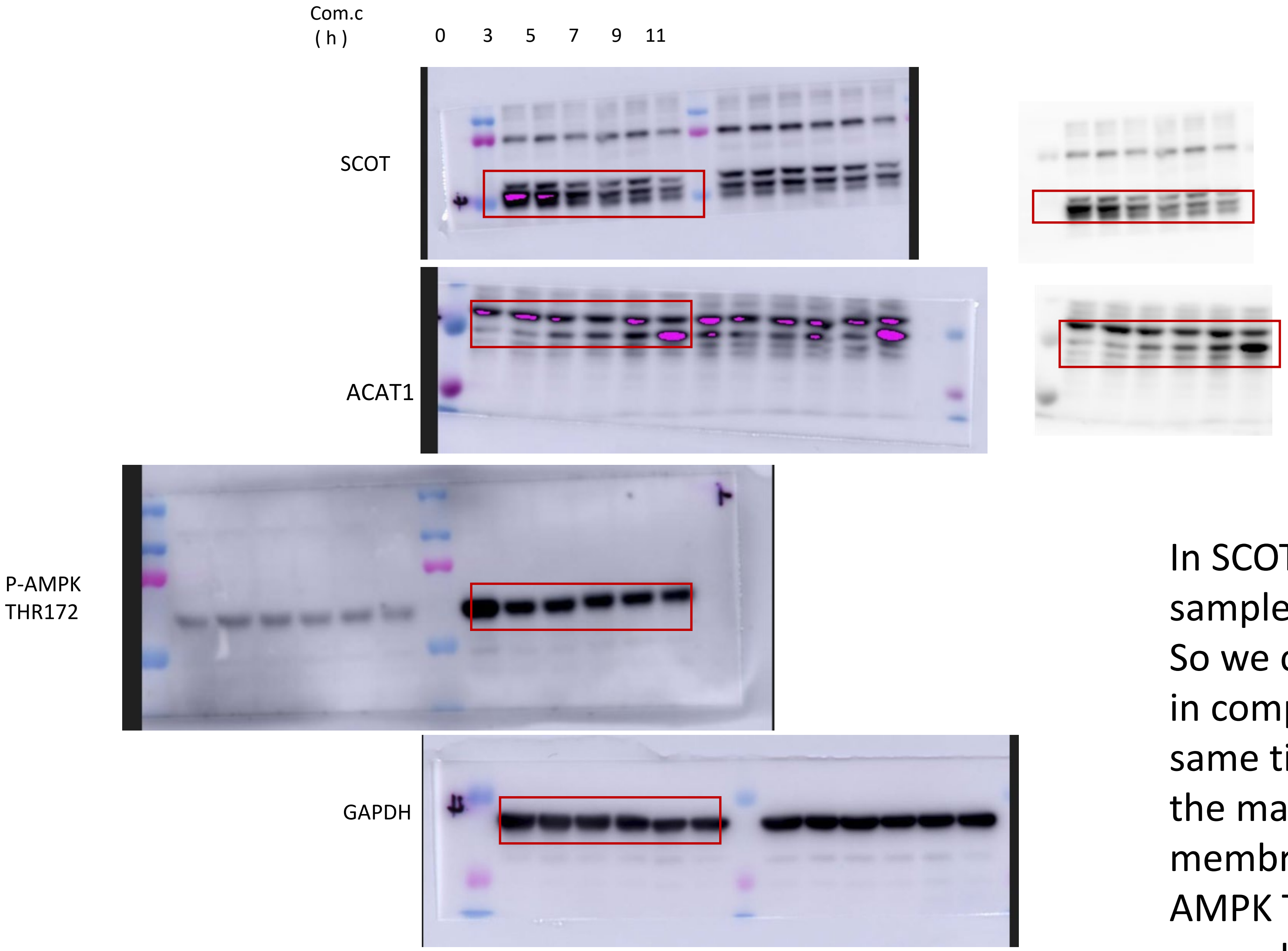

In SCOT, ACAT1 and GAPDH, I use the sample of compound c and A769662. So we can find this membrane is used in compound c and A769662 at the same time. But for ACAT1, I forgot add the marker in the middle. So the membrane is different. For the p-AMPK THR172, because I do it in the second day, the sequence is different with others.

Figure 3D A769662

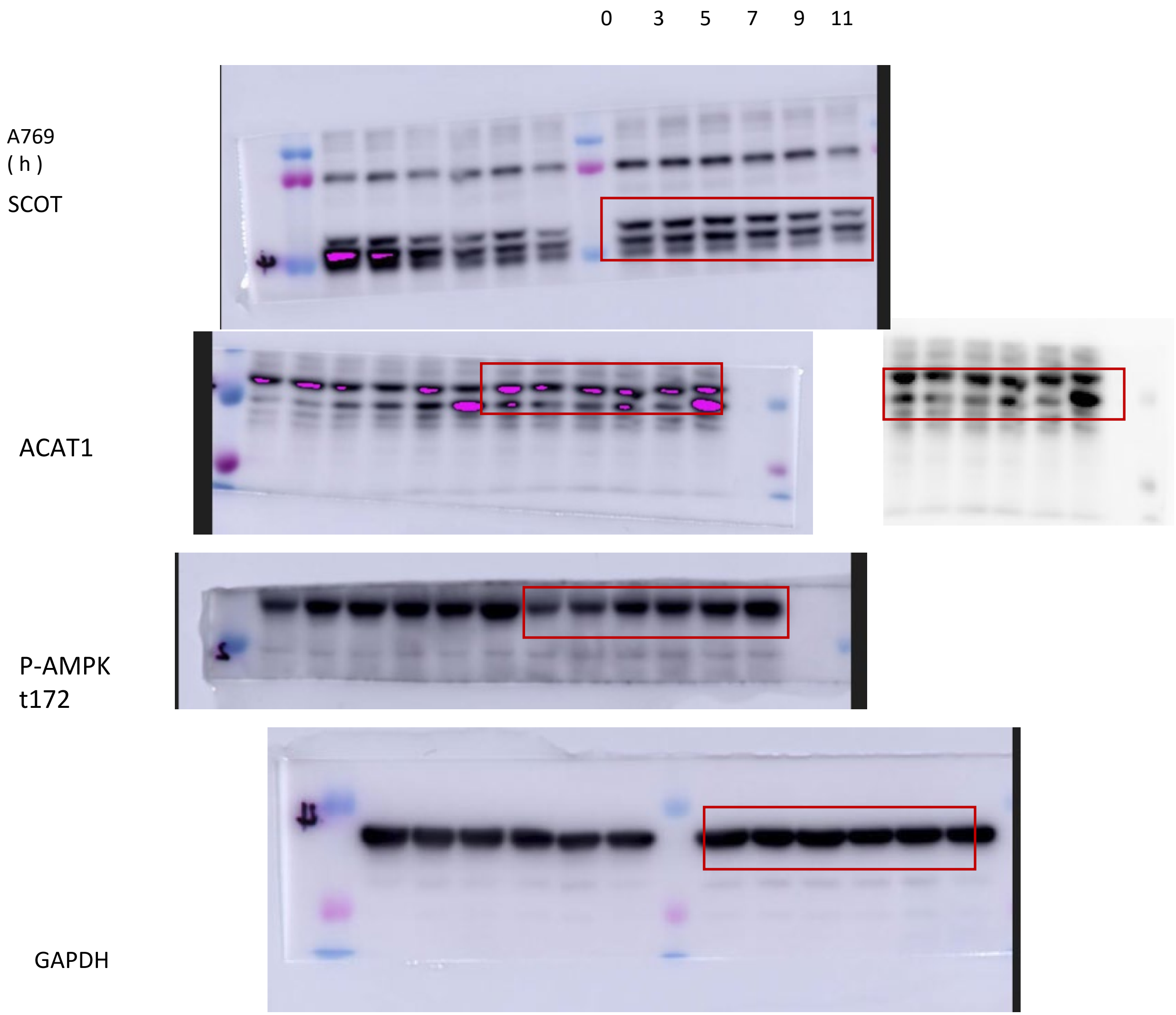

Figure 4D

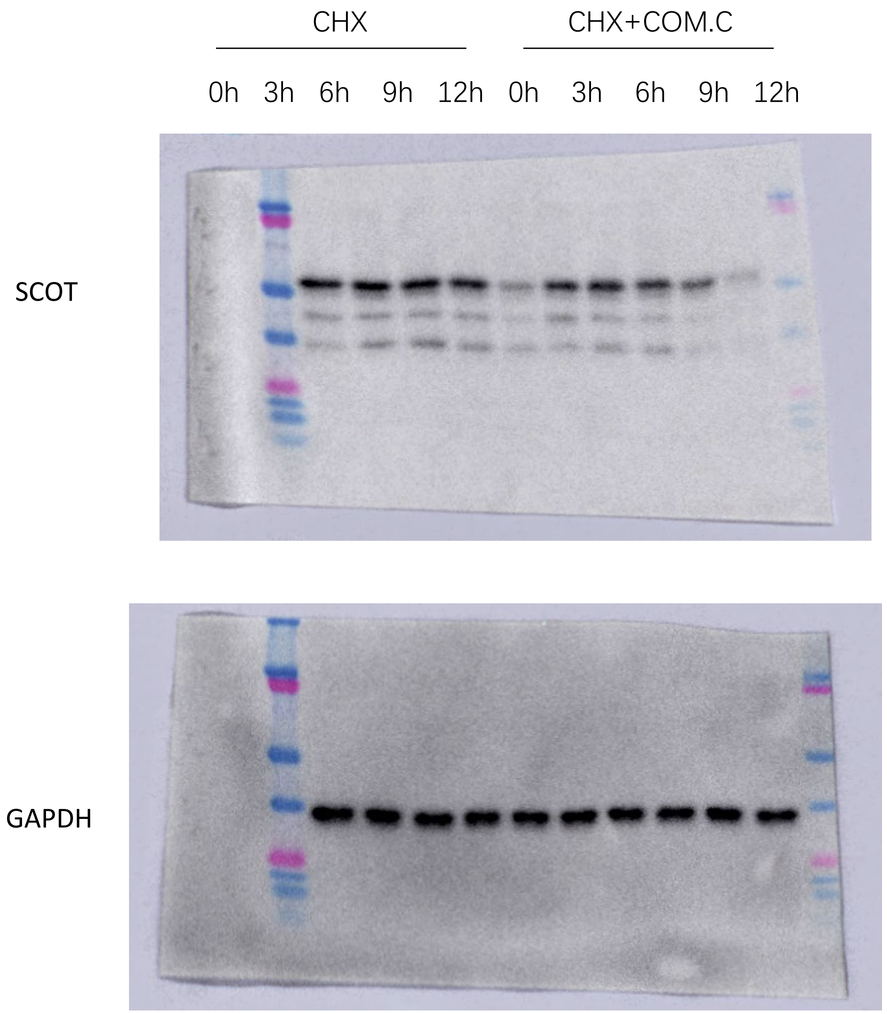

Figure 4E

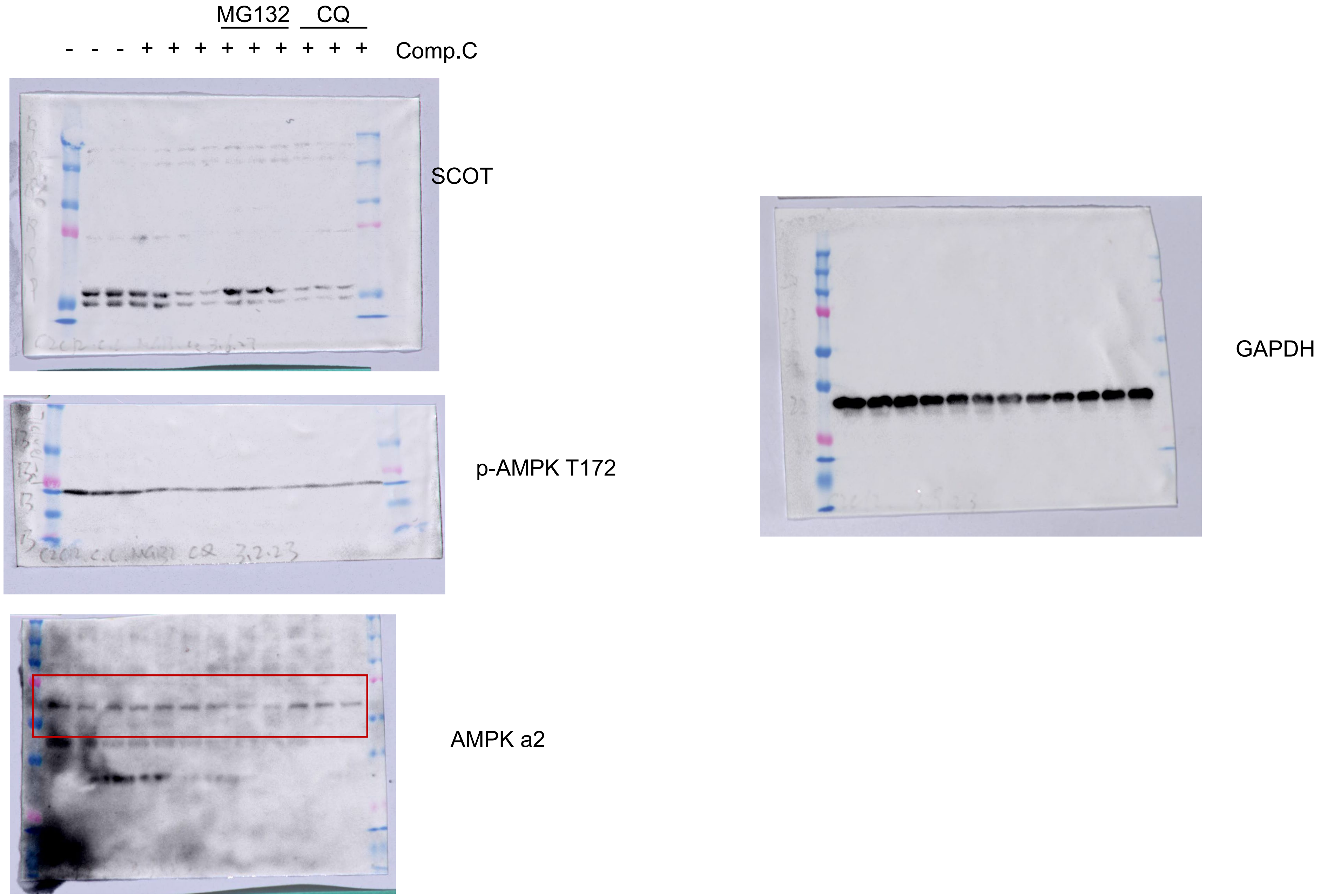

Figure 4F

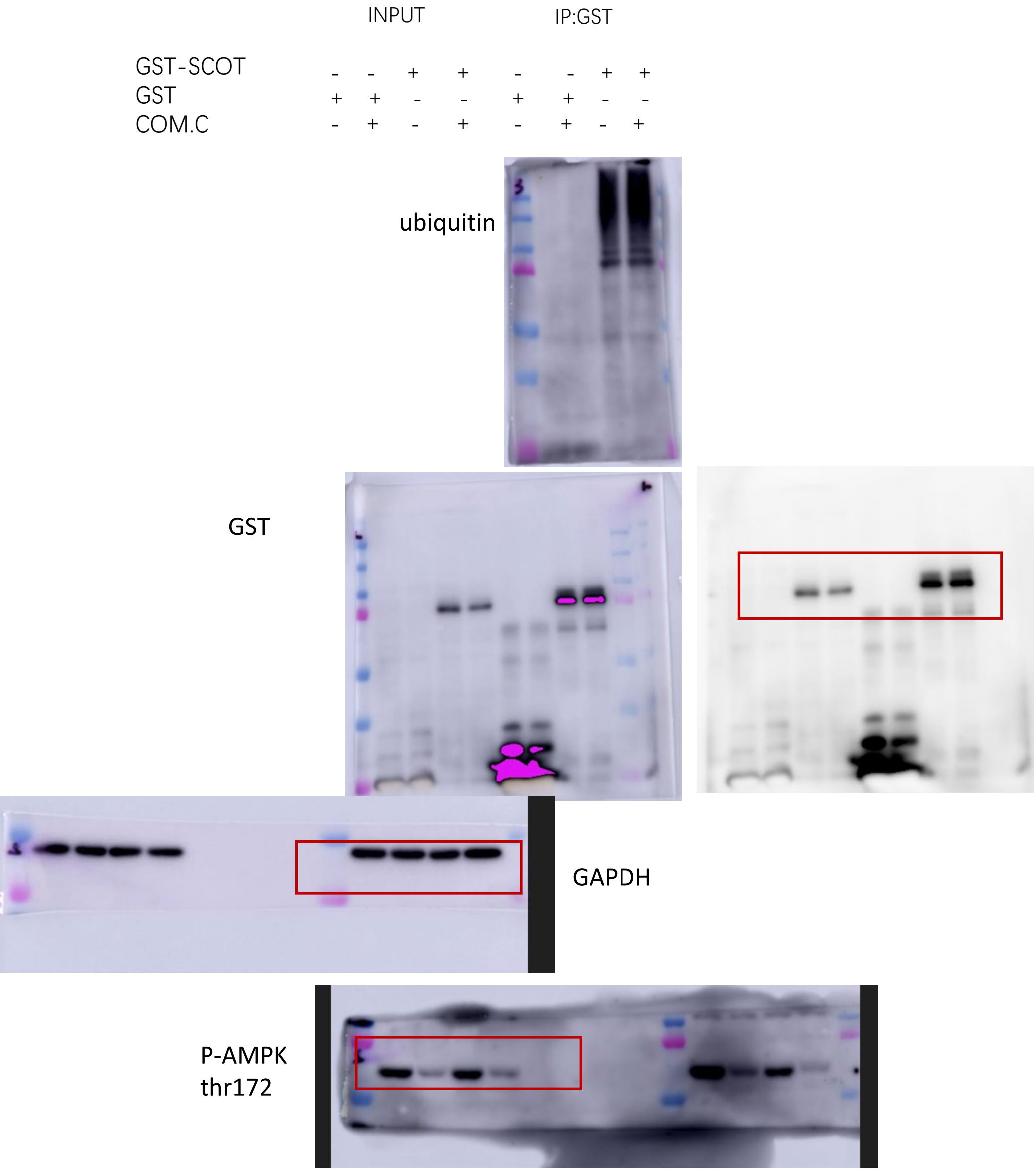
INPUT
IP:GST
GST-SCOT - - + + - - + +
GST + + - - + + - -
COM.C - + - + - + - +
ubiquitin
GST
GAPDH
P-AMPK
thr172

Figure 5A

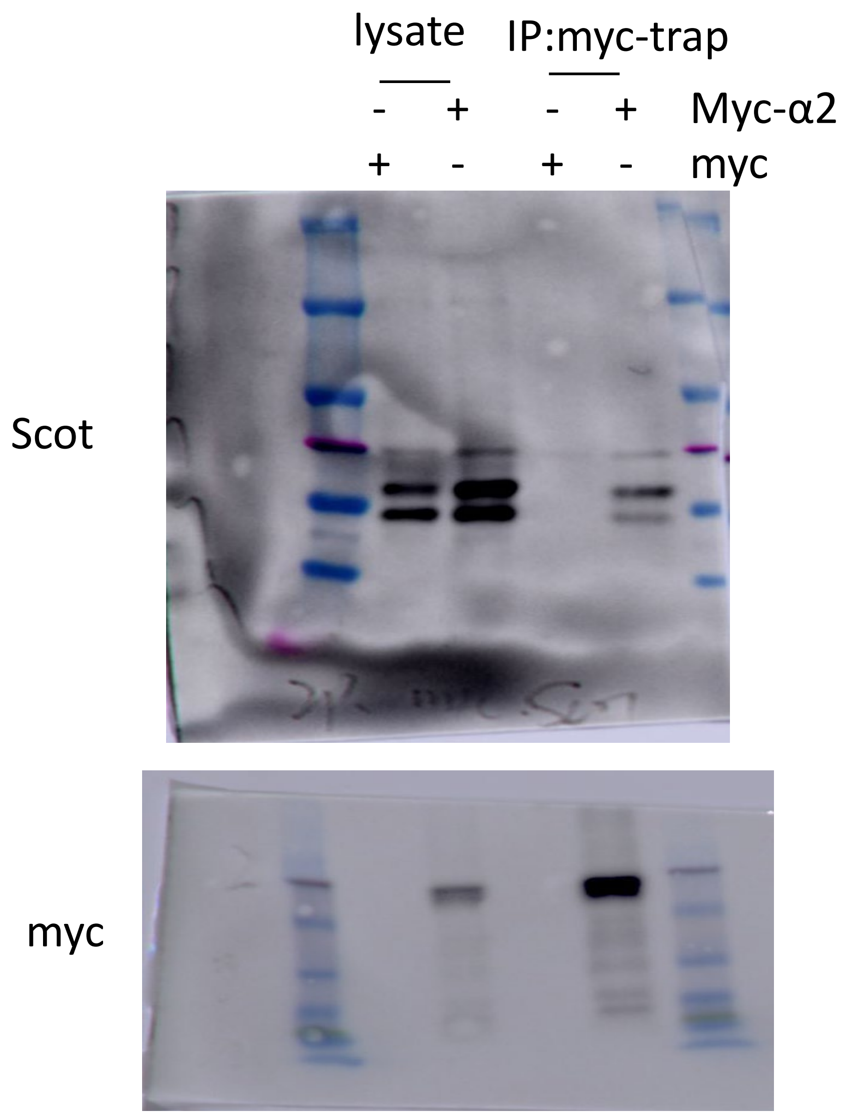

Figure 5B

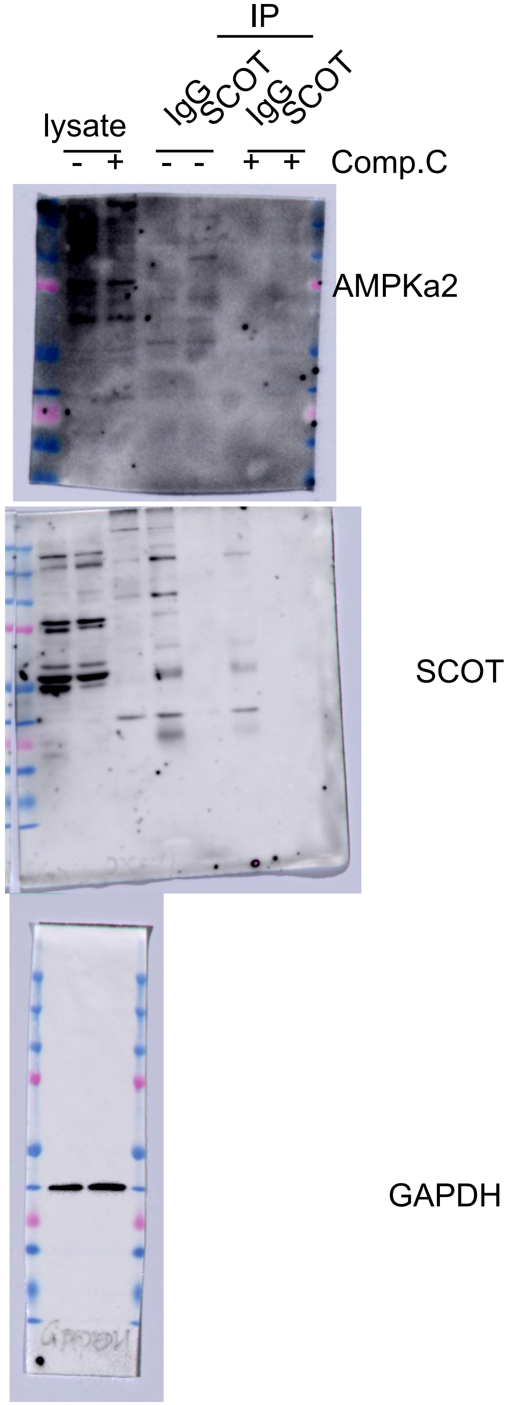

Figure 5C

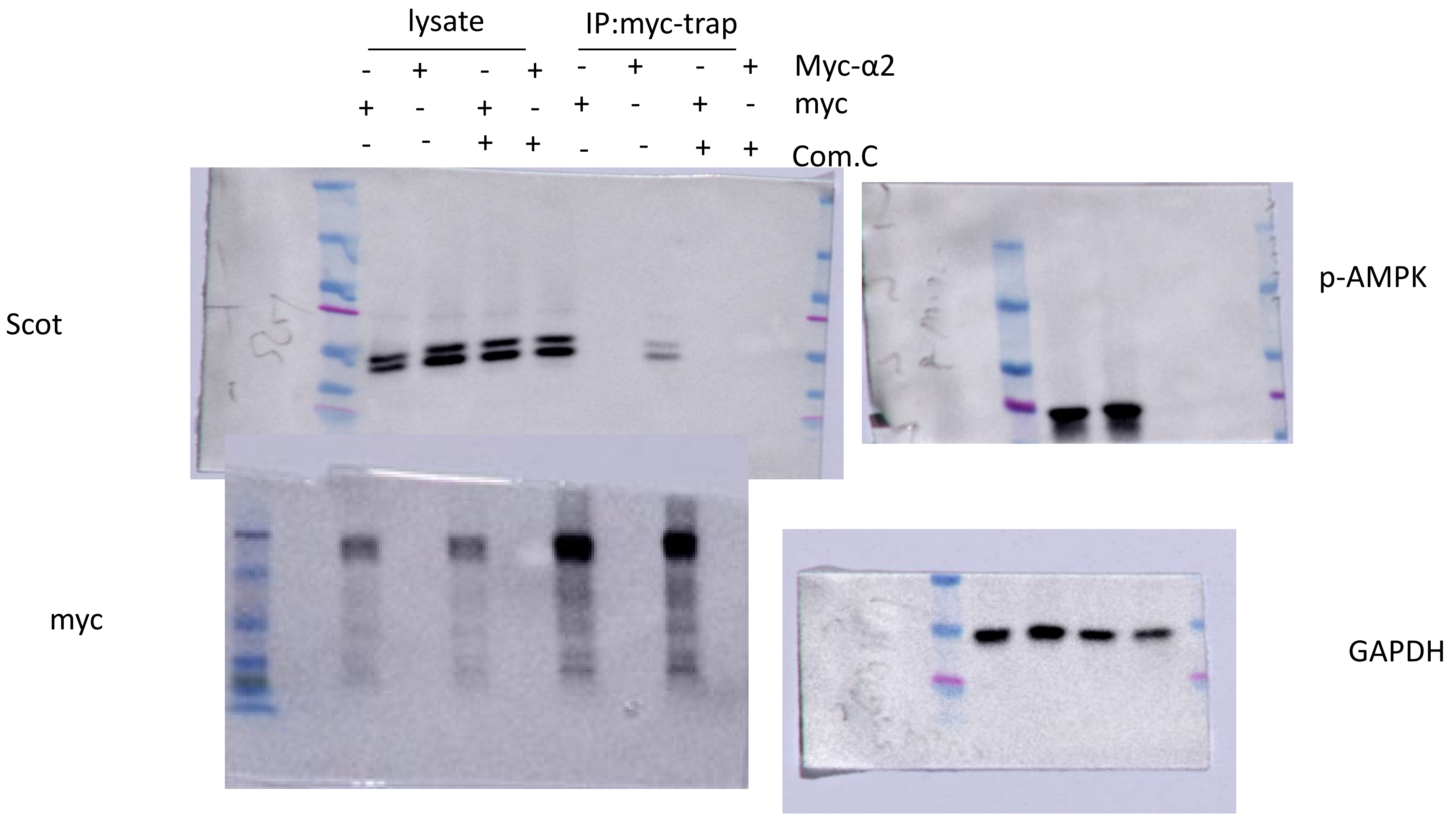

Figure 5D

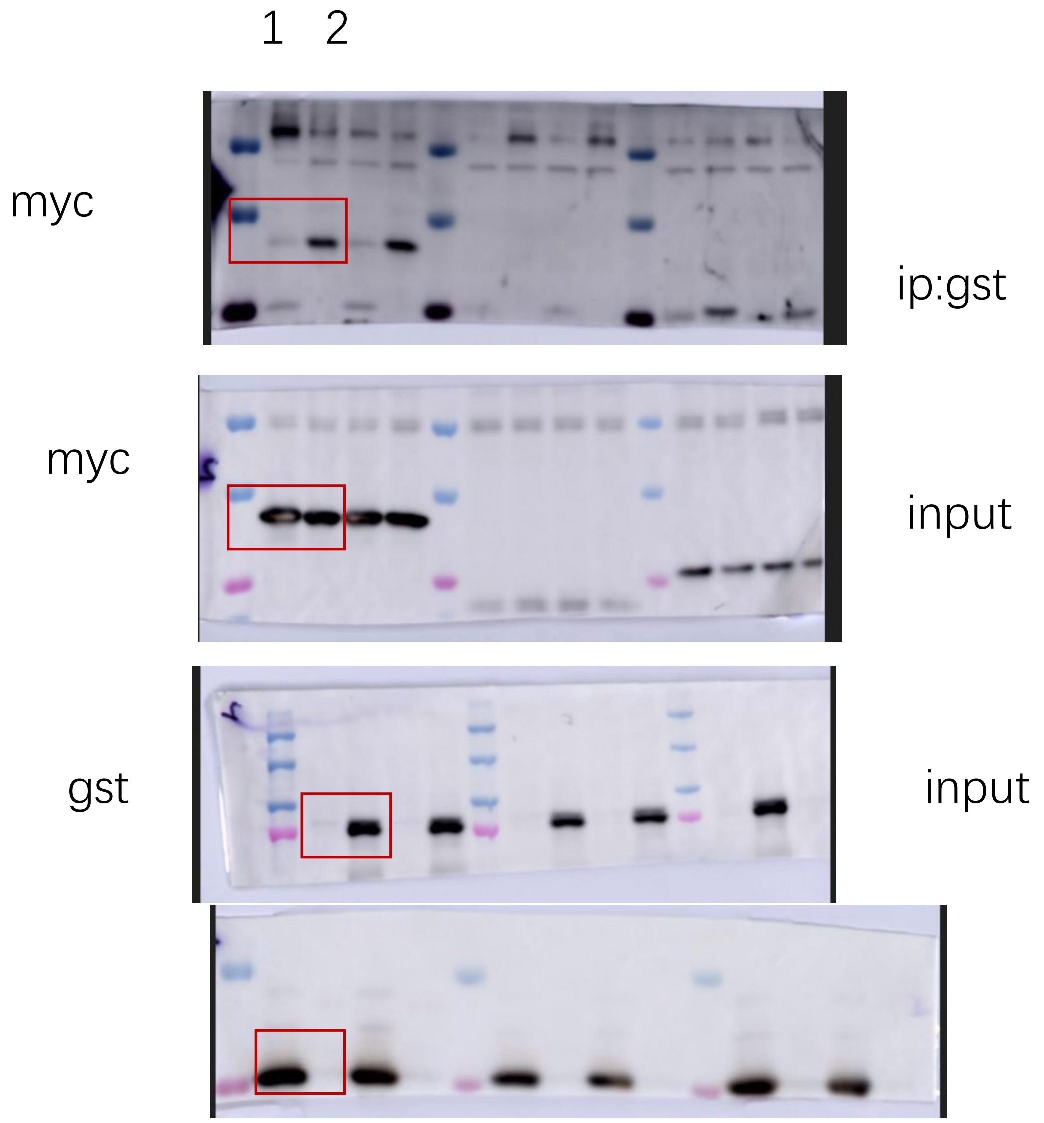

1: GST+ MYC-AMPKα2 domain 1
2: GST-SCOT+ MYC-AMPKα2 domain 1

Figure 5D

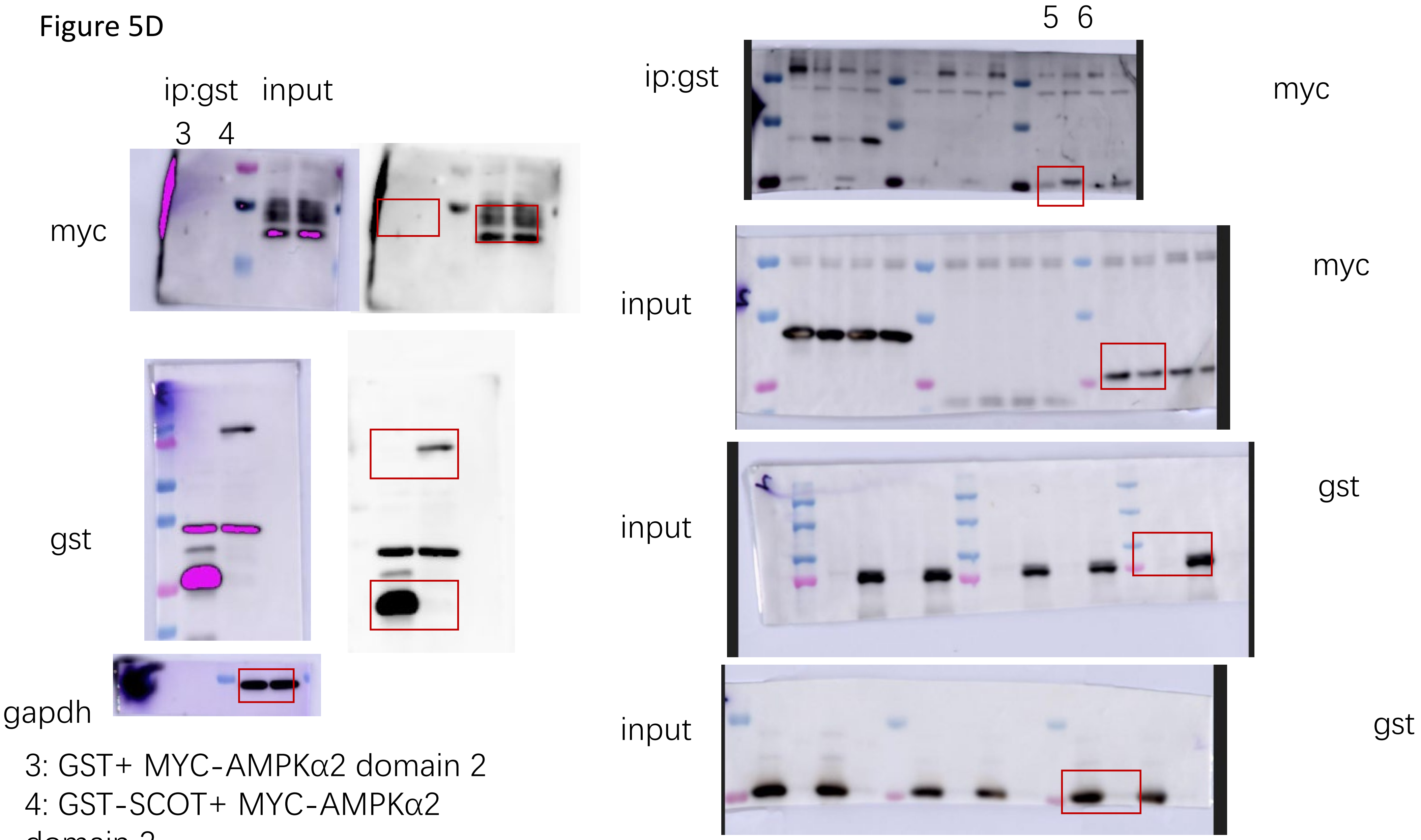

3: GST+ MYC-AMPKα2 domain 2
4: GST-SCOT+ MYC-AMPKα2 domain 2

5: GST+ MYC-AMPKα2 domain 3
6: GST-SCOT+ MYC-AMPKα2 domain 3

Figure 5E

GST-SCOT + MYC- AMPK domain1

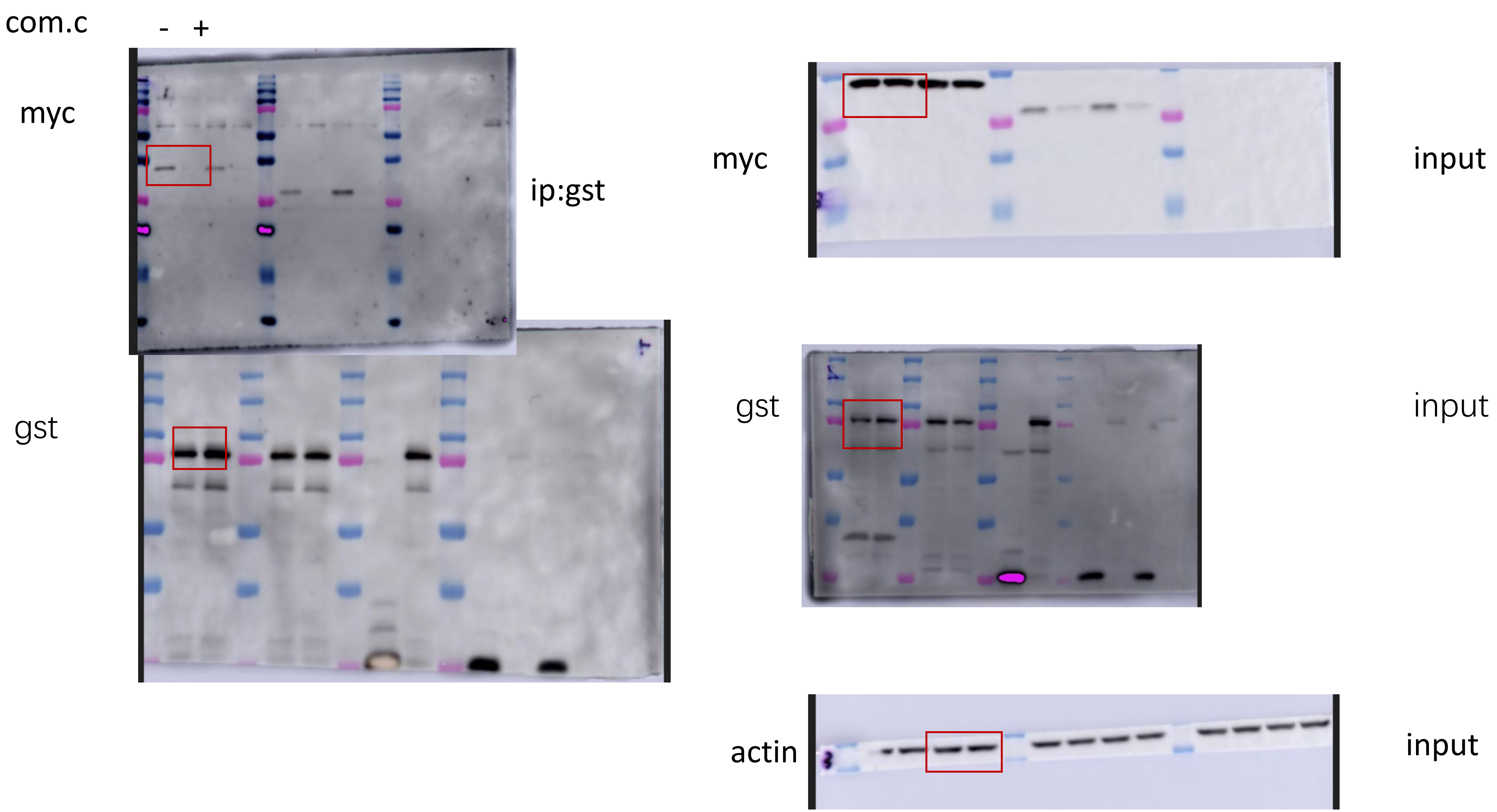

Figure 5F

1: GST-SCOT + AMPKα2 WT
2: GST-SCOT + AMPKα2 K45R

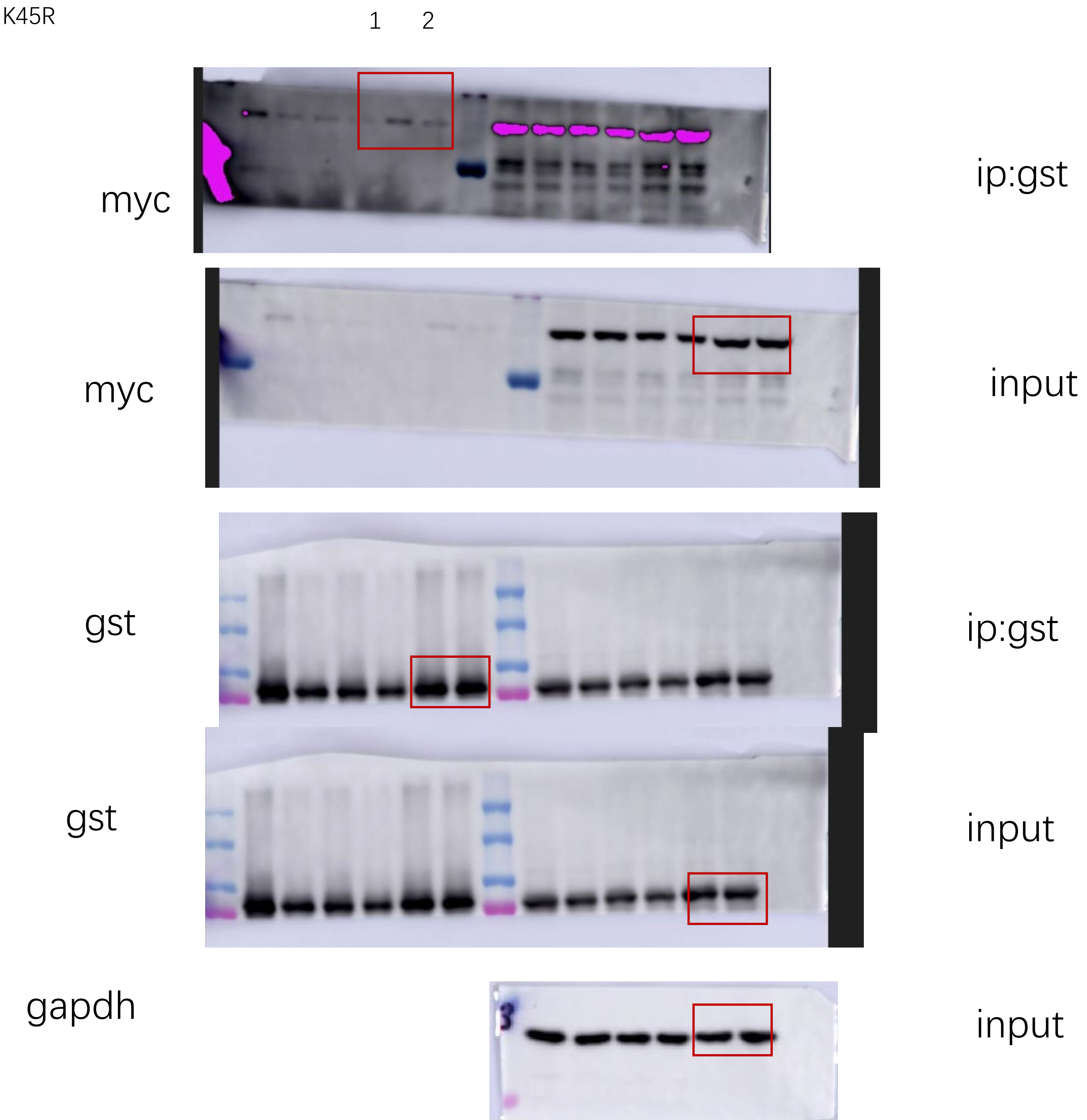

Figure 5G

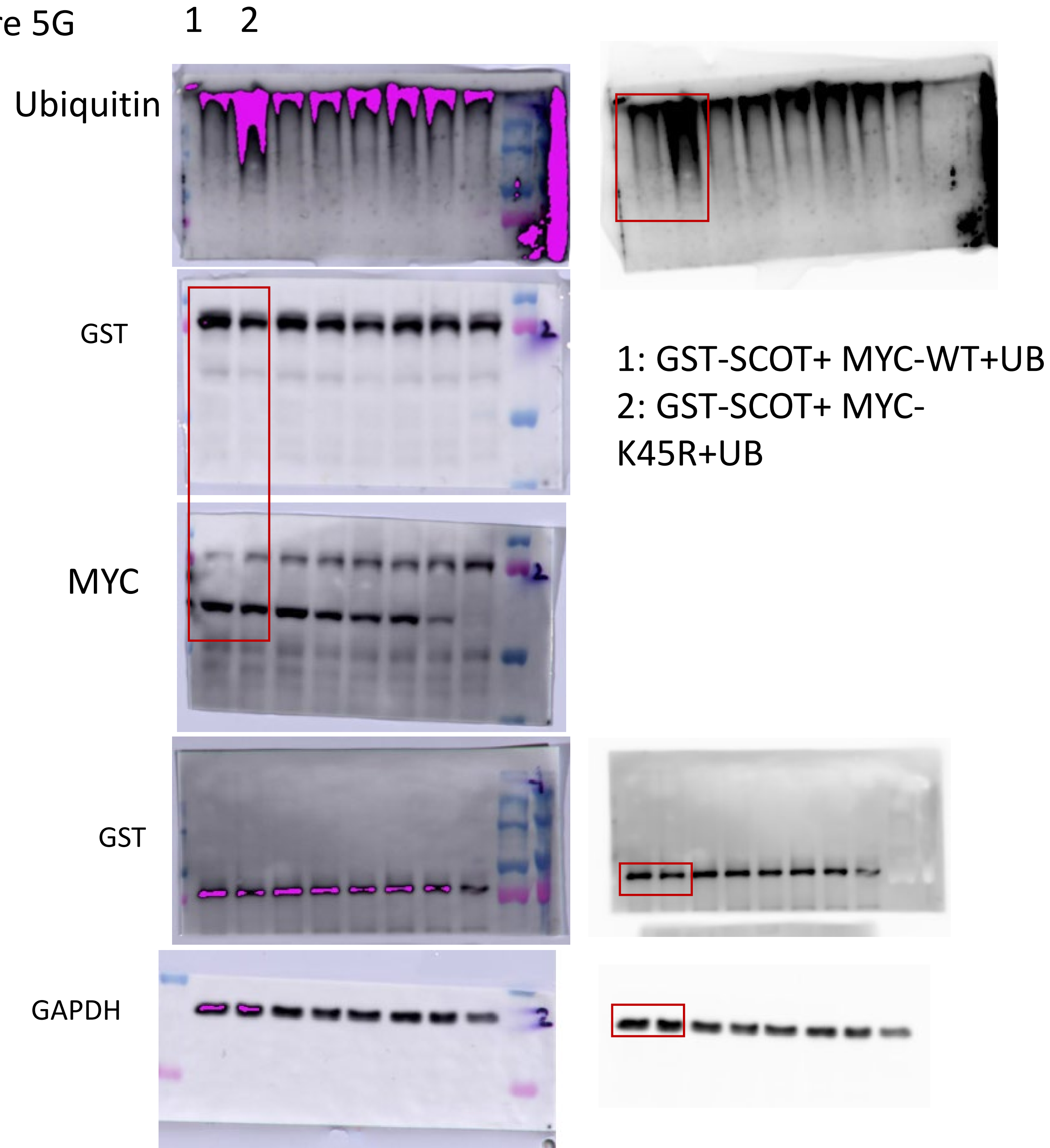
1
2
Ubiquitin
GST
MYC
GST
GAPDH
1: GST-SCOT+ MYC-WT+UB
2: GST-SCOT+ MYC-K45R+UB
